# Vestibular time constant and individual susceptibility to motion sickness in real-world driving

**DOI:** 10.1038/s41598-026-68887-8

**Published:** 2026-09-06

**Authors:** Seonghyeon Kim, Jaesik Yang, Sung Kwang Hong, M. Ercan Altinsoy

**Affiliations:** 1 Chair of Acoustics and Haptics, Institute of Acoustics and Speech Communication, Faculty of Electrical and Computer Engineering, Technische Universität Dresden, Helmholtzstrasse 18, 01069 Dresden, Germany; 2 Research and Development Division, Hyundai Motor Company, Hyundaiyeonguso-ro 150, Hwaseong-si, 18281 Gyeonggi-do South Korea; 3 Department of Otorhinolaryngology–Head and Neck Surgery, Hallym University College of Medicine, Gwanpyeong-ro 170-22, Anyang-si, 14068 Gyeonggi-do South Korea

**Keywords:** Motion sickness, Motion sickness susceptibility, Electric vehicle, Autonomous vehicle, Vestibular time constant, Diseases, Health care, Medical research, Neurology, Neuroscience

## Abstract

Motion sickness is an important challenge for the acceptance of autonomous vehicles. Although the Motion Sickness Susceptibility Questionnaire (MSSQ) is widely used, it relies on retrospective self-report and does not directly capture vestibular characteristics. This study examined the association between the vestibular time constant (VTC), which reflects the velocity storage mechanism, and motion sickness severity during on-road driving. VTC was measured in 24 female adults; 21 completed four 30-min acceleration sessions, and all 24 completed nine 30-min deceleration sessions involving regenerative braking. Motion sickness severity was repeatedly assessed using the Misery Scale (MISC). MSSQ scores showed weak, non-significant associations with MISC-derived outcomes, whereas longer VTCs were associated with greater symptom severity, particularly under deceleration and among participants who developed symptoms. Participant-level receiver operating characteristic analyses showed that the VTC discriminated participants who reached the nausea threshold (MISC ≥ 6) better than the MSSQ. Under deceleration, this difference was significant (area under the curve, 0.89 versus 0.60; DeLong *p* = 0.048). VTC associations were descriptively stronger under deceleration, possibly reflecting condition-specific characteristics of braking-related vestibular stimulation. These findings indicate that the VTC provides objective physiological information that may complement questionnaire-based susceptibility assessment in automotive environments.

**Supplementary Information:**

The online version contains supplementary material available at https://doi.org/10.1038/s41598-026-68887-8.

## Introduction

Autonomous driving technology is transforming mobility by reducing driving demands and enabling occupants to engage in non-driving-related activities (NDRAs) such as work, leisure, and rest. However, compared with conventional driving, automated driving may increase the risk of motion sickness because occupants have less control over vehicle motion and may be less able to anticipate upcoming movements^1^. Motion sickness is commonly attributed to conflicts among visual, vestibular, and somatosensory inputs, which can lead to symptoms such as discomfort and nausea^2,3^. This risk may be particularly pronounced when passengers engage in NDRAs, such as using smartphones or laptops, rather than viewing the road ahead, thereby increasing the potential for visual–vestibular conflict^4,5^. Sivak and Schoettle^6^ estimated that 6–10% of American adults travelling in fully automated vehicles would often, usually, or always experience some degree of motion sickness. Collectively, visual–vestibular conflict, reduced motion predictability, and lack of control over vehicle dynamics make motion sickness an important challenge for the acceptance and use of automated vehicles.

Various pharmacological and non-pharmacological strategies have been investigated to prevent or mitigate motion sickness in automated vehicles. Pharmacological interventions, including antihistamines and vestibular suppressants, can reduce motion sickness symptoms, but their potential side effects motivate the development of non-pharmacological alternatives^7^. More recently, transcutaneous auricular vagus nerve stimulation (taVNS) has emerged as a promising non-pharmacological approach to visually induced motion sickness. Recent randomized sham-controlled studies showed that taVNS reduced motion sickness symptoms, with convergent evidence from cortical activity assessed using exact low-resolution brain electromagnetic tomography (eLORETA) and autonomic dynamics assessed using electrocardiogram-based symmetric projection attractor reconstruction (SPAR)^8,9^. Other non-pharmacological approaches include increasing visibility of the external environment and providing intuitive information about upcoming vehicle motion, which may reduce visual–vestibular conflict^10^. Anticipatory visual, auditory, and vibrotactile cues have also been investigated as a means of improving motion predictability and alleviating symptoms^11–15^. Repeated exposure may further promote habituation to motion stimuli over time^16^. Vehicle-based approaches include trajectory optimization and smoother acceleration and deceleration profiles designed to reduce provocative motion stimuli^17–19^. Improvements in seat stability and occupant posture may provide additional benefits by reducing unnecessary vestibular stimulation^20,21^.

Despite these mitigation efforts, susceptibility to motion sickness varies substantially among individuals, with some developing severe symptoms under conditions that have little or no effect on others^22^. Previous studies have associated this variability with demographic, physiological, and psychological factors, including age, sex, prior motion exposure, and individual expectations^16,22^. Women have generally been reported to show greater susceptibility than men, although the mechanisms underlying this difference are likely multifactorial and remain incompletely understood^23^. To characterize such individual differences, the Motion Sickness Susceptibility Questionnaire (MSSQ) is widely used as a standardized assessment tool. The MSSQ estimates susceptibility from recalled motion sickness experiences across different motion environments and provides a reliable and practical self-report measure^16,22,24^. However, because it relies on retrospective self-report, MSSQ scores may be influenced by recall accuracy and previous exposure to the motion environments assessed. Moreover, the questionnaire does not directly measure physiological characteristics that may contribute to motion sickness susceptibility, potentially limiting its applicability to novel driving environments.

To complement questionnaire-based assessments, the vestibular time constant (VTC) has been investigated as a potential physiological marker of motion sickness susceptibility. The VTC characterizes the decay of the vestibulo-ocular response following rotational stimulation and reflects the velocity storage mechanism, which prolongs vestibular responses beyond the dynamics of the semicircular canals^25–27^. Velocity storage has both temporal and spatial properties. Dai et al.^28^ showed that the spatial orientation of the stored velocity signal is influenced by gravity and may differ from the direction of the evoked eye-velocity response. Such spatial misalignment may contribute to the sensory conflict implicated in motion sickness^26,27^. A longer VTC indicates greater persistence of vestibular signals and may increase the potential for sensory conflict during motion^29,30^. Several studies support an association between the VTC and motion sickness susceptibility. Individuals with bilateral vestibular deficits, who generally exhibit shorter time constants, have shown reduced susceptibility to motion sickness^31^. Visual–vestibular training has also been associated with both a reduction in the VTC and a prolonged reduction in motion sickness susceptibility^32^. More recently, Lagami et al.^33^ reported that individuals with shorter VTC values were less susceptible to seasickness induced predominantly by vertical heave motion.

The motion characteristics of maritime environments differ from those encountered in road vehicles, where longitudinal and lateral accelerations, rotational motion, and transient changes in vehicle dynamics may occur simultaneously. Experimental setting may also influence the manifestation of motion sickness. Talsma et al.^34^ compared motion sickness experienced in a moving-base driving simulator with that experienced during on-road driving. Although symptom profiles were correlated between the two environments, gastrointestinal symptoms were more prominent during on-road driving, whereas central and sopite-related symptoms were more prominent in the simulator. These findings indicate that results obtained under laboratory or maritime conditions may not directly generalize to motion sickness experienced in road vehicles.

Despite this evidence, empirical studies directly examining the association between the VTC and motion sickness during on-road driving remain limited. Most previous investigations have evaluated the VTC in laboratory or maritime settings, which may not fully represent the transient and multidirectional motion stimuli encountered in road vehicles. It therefore remains unclear whether the VTC is associated with motion sickness severity under on-road driving conditions and how its discriminative ability compares with that of the MSSQ.

Accordingly, this study investigated the association between the VTC and motion sickness severity during controlled on-road driving and compared its discriminative ability with that of the MSSQ. VTC was measured in 24 participants, who completed up to 13 separate 30-min driving sessions comprising four acceleration profiles and nine regenerative-braking deceleration profiles. Motion sickness severity was assessed at regular intervals using the Misery Scale (MISC). Associations among VTC values, MSSQ scores, and MISC-derived outcomes were examined using correlation analyses, and participant-level receiver operating characteristic (ROC) analyses were conducted to compare the ability of the VTC and the MSSQ to discriminate participants who reached the nausea threshold.

## Related work

### Motion sickness susceptibility questionnaire

The MSSQ is a retrospective self-report instrument used to assess individual susceptibility to motion sickness across different motion environments. The revised long-form MSSQ developed by Golding^16^, which was used in the present study, assesses previous experiences of motion sickness in common forms of transportation and other motion-provoking environments, including cars, buses, trains, aircraft, boats, swings, and amusement rides. The questionnaire comprises two sections: Part A assesses motion sickness experiences during childhood before the age of 12, whereas Part B assesses experiences during the preceding 10 years of adulthood. Respondents provide ordinal ratings of their previous motion sickness experiences for each applicable motion environment, and the responses are combined to produce childhood, adulthood, and total susceptibility scores. The scoring procedure accounts for motion environments that the respondent has not experienced.

The MSSQ provides a reliable and practical measure of trait susceptibility without exposing individuals to provocative motion^16,22,24^. It has therefore been widely used to characterize individual differences and recruit participants for motion sickness research. However, MSSQ scores depend on retrospective recall and previous exposure to the motion environments included in the questionnaire. Differences in exposure history and recall accuracy may consequently influence the resulting scores. Moreover, the MSSQ does not directly assess physiological characteristics that may contribute to susceptibility, and its association with symptom severity may vary across motion environments. In the present study, the MSSQ was therefore used as a questionnaire-based reference measure against which the association and discriminative ability of the VTC were compared.

### Misery scale

The MISC is an 11-point single-item ordinal scale used to assess the current severity of motion sickness in experimental and on-road environments^35^. Scores range from 0 to 10 and represent a progression of motion sickness symptoms: 0 indicates no problems, 1 indicates uneasiness without specific symptoms, and scores of 2–5 indicate increasing severity of symptoms such as dizziness, warmth, headache, stomach awareness, and sweating without nausea. Scores of 6–8 represent slight to severe nausea, whereas 9 indicates near retching and 10 indicates vomiting. Thus, a score of 6 represents the nausea threshold. Because the MISC can be administered rapidly and repeatedly, it is suitable for monitoring changes in symptom severity during continuous motion exposure.

Unlike the MSSQ, which retrospectively assesses trait susceptibility, the MISC provides a state-based assessment of symptoms at the time of measurement. It has therefore been widely used to monitor the temporal development of motion sickness during vehicle, simulator, and visually induced motion exposures. However, the MISC remains a subjective measure and may be influenced by individual differences in symptom perception and reporting criteria. Moreover, as a single ordinal rating, it summarizes symptom progression rather than separately quantifying individual symptom domains. In the present study, repeated MISC ratings were used to characterize the development and severity of motion sickness during the two on-road driving conditions.

### Vestibular time constant

The VTC is a physiological measure that characterizes the decay of the vestibulo-ocular response following rotational stimulation. It reflects the central velocity storage mechanism, which prolongs and spatially integrates vestibular signals beyond the peripheral dynamics of the semicircular canals^25,26^. This mechanism improves the encoding of low-frequency rotational motion and contributes to spatial orientation. Operationally, the VTC is defined as the time required for the slow-phase velocity of the vestibular response to decay to approximately 37% of its initial value^32^. A longer VTC therefore indicates greater persistence of the velocity storage response.

The temporal persistence of vestibular signals may contribute to motion sickness when vestibular information is inconsistent with visual or somatosensory input. Longer VTC values have been associated with greater motion sickness susceptibility, whereas shorter values have been observed in individuals with bilateral vestibular deficits and lower susceptibility^26,31,36^. Pharmacological modulation of velocity storage with baclofen has also been investigated as a means of reducing motion sickness susceptibility^37^. These findings support the physiological relevance of the VTC, although its relationship with symptom severity may depend on the characteristics of the motion environment.

The VTC is commonly estimated using a rotary-chair step-velocity test conducted in darkness to minimize visual fixation. Following a period of constant-velocity rotation, chair deceleration elicits post-rotatory nystagmus, which can be recorded using electrooculography or video-oculography^38,39^. Nystagmus consists of a vestibularly driven slow phase and a compensatory fast phase^40,41^. The VTC is estimated by fitting an exponential decay function to the slow-phase velocity after rotation. This procedure provides a quantitative measure of the persistence of the velocity storage response. VTC values vary among individuals and may be influenced by age, vestibular function, and measurement conditions; therefore, they should be interpreted in the context of the participant population and testing protocol.

## Methods

**Fig. 1:**
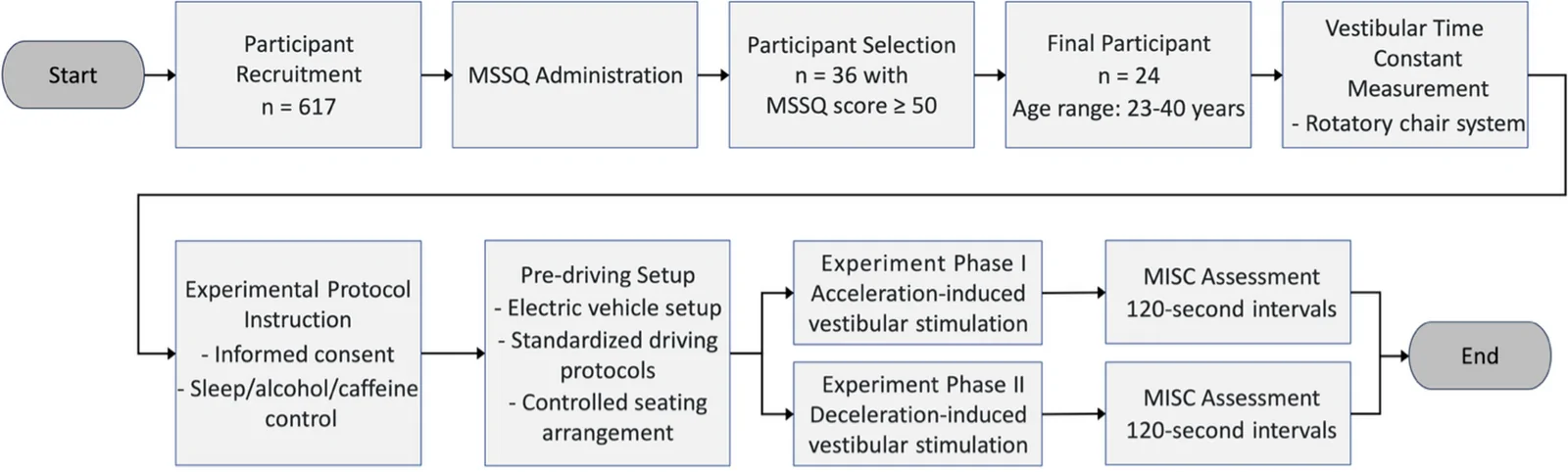
Overview of the study procedure, including participant recruitment, VTC measurement, and controlled on-road driving under acceleration- and deceleration-dominant conditions. MISC ratings were obtained at regular intervals during each condition. MSSQ, Motion Sickness Susceptibility Questionnaire; VTC, vestibular time constant; MISC, Misery Scale.

An overview of the study procedure, including participant recruitment, VTC measurement, and the two controlled on-road driving conditions, is presented in Fig. 1.

### Participants

Based on previous evidence that women show, on average, greater susceptibility to motion sickness than men^42^, recruitment was restricted to adult women to increase the likelihood of obtaining measurable symptom responses during the on-road protocol. A total of 617 individuals completed the long-form MSSQ screening survey. Of these, 36 individuals with normal or corrected-to-normal vision and a raw long-form MSSQ score of 50 or higher were invited to participate. This pragmatic eligibility threshold was slightly above the published population mean of 45.5 (SD 37.6) and was intended to recruit individuals with at least average motion sickness susceptibility^16^. The 36 invited individuals had a mean age of 32.5 years (SD 5.6). Twelve withdrew for personal reasons before data collection, resulting in a final sample of 24 participants with a mean age of 33.2 years (SD 5.5).

All 24 participants completed the VTC assessment and Experiment 2. Three participants were unable to participate in Experiment 1 because of poor general condition on the scheduled test day, unrelated to the motion sickness protocol. They were therefore excluded from the Experiment 1 analyses, which were based on complete data from 21 participants. These three participants subsequently participated in Experiment 2, for which data from all 24 participants were analyzed. Participants who developed motion sickness during Experiment 1, including those who reached the nausea threshold, were retained in the analyses.

Before participation, all participants received a detailed explanation of the study procedures and provided written informed consent. They were instructed to obtain at least 6 h of sleep before each session, refrain from consuming alcohol or caffeine for 24 h before testing, and temporarily discontinue medication use where medically appropriate and under the guidance of their healthcare provider. To reduce menstrual-cycle-related variability in vestibular sensitivity and motion sickness susceptibility, testing was scheduled outside the self-reported menstrual phase for all participants. The study protocol was approved by the Institutional Review Board of Hallym University Sacred Heart Hospital, South Korea (IRB No. 2023-08-003-001), and all procedures were conducted in accordance with the Declaration of Helsinki.

### Vestibular time constant measurement

The VTC was measured using a clinical rotary-chair system (CHARTR 200; Natus Medical ApS, Denmark) enclosed within a cylindrical structure to eliminate external visual input and provide complete darkness. The assessment was conducted in the Department of Otorhinolaryngology at Hallym University Sacred Heart Hospital, South Korea. Each participant’s head was positioned and securely restrained to align the lateral semicircular canals with the plane of chair rotation. Participants were instructed to remain alert throughout the examination.

Horizontal eye movements were recorded using electro-oculography (EOG). Because the VTC was obtained as part of the hospital’s clinical vestibular examination, the EOG modality integrated into the rotary-chair system was used. Previous work comparing eye-movement recording methods during rotary-chair testing found no significant difference between EOG- and video-oculography (VOG)-derived mean slow-phase velocities for the horizontal vestibulo-ocular reflex^43^. Electrodes were positioned to record horizontal nystagmus in complete darkness. The clinical examination included sinusoidal harmonic acceleration (SHA) and step-velocity tests. During the SHA test, the chair oscillated about the yaw axis at 0.01, 0.02, 0.04, 0.08, 0.16, 0.32, and 0.64 Hz, with a peak angular velocity of 50 $\:^\circ\:/s$. The SHA test was used to evaluate vestibulo-ocular reflex dynamics across frequencies, whereas the step-velocity test was used to estimate the VTC.

During the step-velocity test, the chair accelerated from rest at 100°/s² for 1 s, maintained a constant angular velocity of 100 $\:^\circ\:/s$ for 60 s, and then decelerated at − 100 $\:^\circ\:/{s}^{2}$ for 1 s to a stop. The test was performed in both clockwise and counterclockwise directions. Slow-phase eye velocity during the per-rotatory and post-rotatory responses was fitted with an exponential decay function. The time constant was defined as the time required for the fitted response to decrease to approximately 37% of its initial amplitude, following established method^44^. Four time constants were obtained from the per-rotatory and post-rotatory responses during rightward and leftward chair rotations. The time constant for right-beating nystagmus was calculated by averaging the right per-rotatory and left post-rotatory responses, whereas the time constant for left-beating nystagmus was calculated by averaging the left per-rotatory and right post-rotatory responses. A single participant-level VTC was then calculated as the mean of these two directional time constants and used in the subsequent analyses. EOG was used only during the rotary-chair examination and was not recorded during the 30-min on-road driving sessions.

### Experimental apparatus and condition

Motion sickness was evaluated under controlled on-road driving conditions using two identically configured electric vehicles (EV6 23MY; Kia, South Korea). Participants were randomly assigned to one of the two vehicles, both of which were operated by professional drivers following the same predefined driving profiles. Participants and an experimenter were seated in the rear passenger seats. A tablet PC was mounted in front of each participant for a non-driving-related task (NDRT), which consisted of watching videos during each driving session. Every 2 min, the experimenter prompted participants to rate their current motion sickness severity using the MISC, and participants entered their ratings on a separate tablet device. Curtains were installed between the front and rear rows and over the windows to restrict participants’ view of the external road environment.

**Fig. 2:**
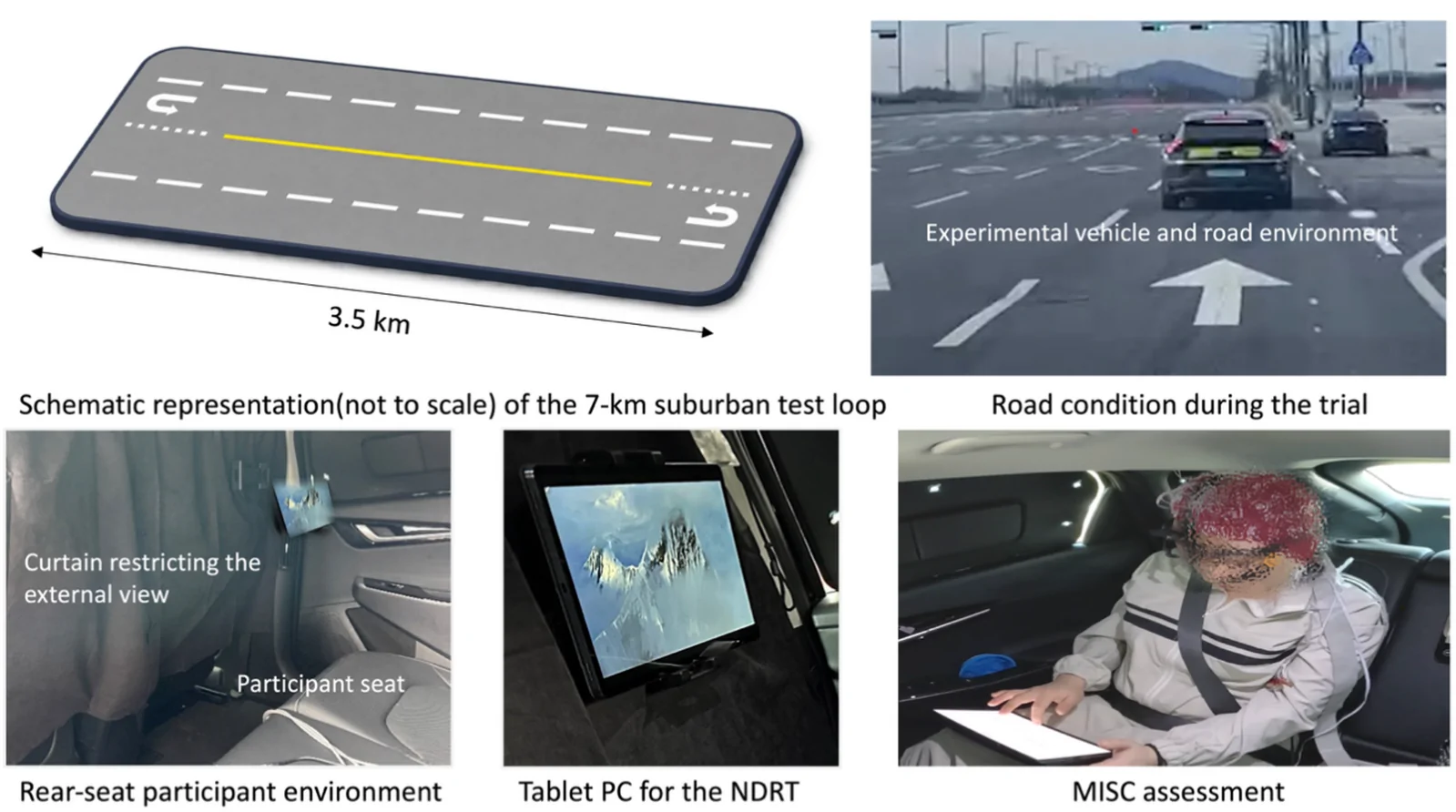
Experimental setup and 7-km on-road test course. The rear-seat environment included curtains restricting the external view, a tablet-based NDRT, and tablet-based MISC reporting. NDRT, non-driving-related task; MISC, Misery Scale.

The driving sessions were conducted on public suburban roads in Gyeonggi-do, South Korea (37.25° N, 126.77° E). The test course consisted of a 7-km loop that included longitudinal vehicle motion, including acceleration, constant-speed driving, and deceleration, as well as lateral motion during turning maneuvers. The loop was driven repeatedly during each 30-min session. Figure 2 presents the experimental setup and test course.

**Fig. 3:**
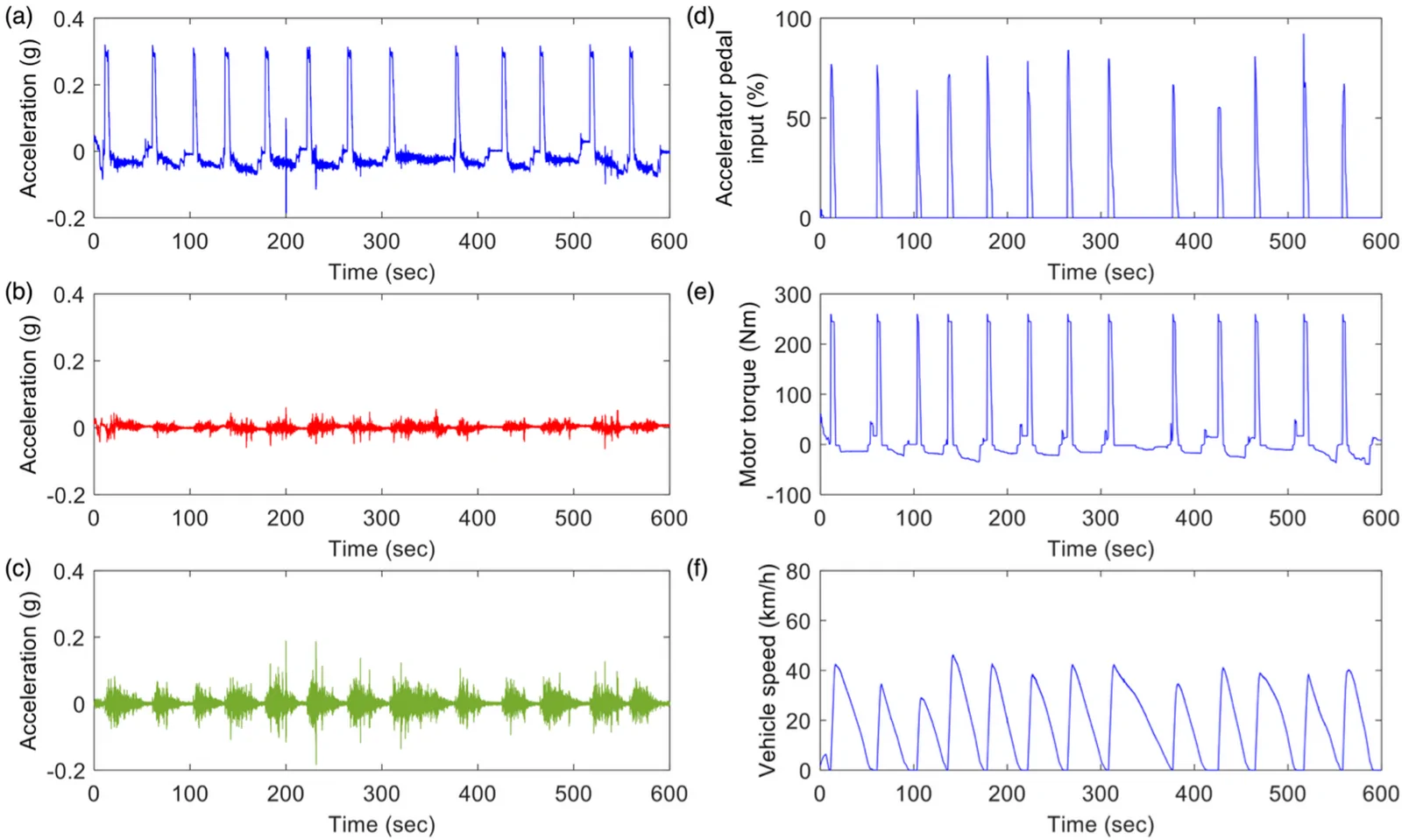
Representative vehicle dynamics during the acceleration-induced condition. Time histories of longitudinal acceleration (**a**), lateral acceleration (**b**), and vertical acceleration (**c**), as well as accelerator pedal input (**d**), motor torque (**e**), and vehicle speed (**f**).

**Fig. 4:**
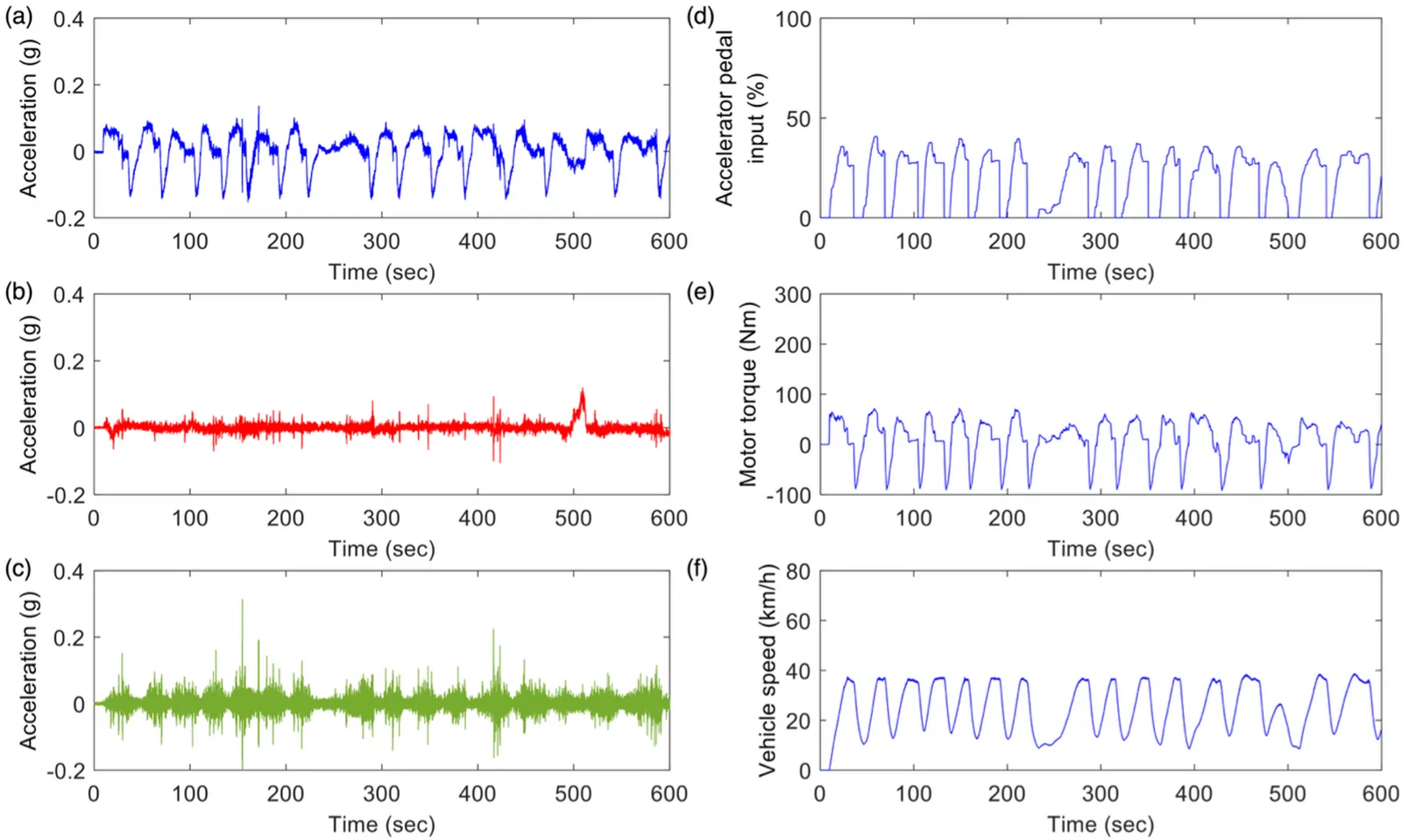
Representative vehicle dynamics during the deceleration-induced condition with regenerative braking. Time histories of longitudinal acceleration (**a**), lateral acceleration (**b**), and vertical acceleration (**c**), as well as accelerator pedal input (**d**), motor torque (**e**), and vehicle speed (**f**).

Two longitudinal-motion conditions were evaluated. Experiment 1 was an acceleration-induced condition characterized by rapid acceleration, constant-speed driving, and gradual deceleration. Experiment 2 was a deceleration-induced condition characterized by gradual acceleration, constant-speed driving, and deceleration generated primarily through regenerative braking. Representative time histories of vehicle acceleration, accelerator pedal input, motor torque, and vehicle speed under the two conditions are presented in Figs. 3 and 4. Because longitudinal acceleration changed linearly toward the predefined target, nominal ramp jerk magnitude was calculated as the absolute change in longitudinal acceleration divided by the time to target. Experiment 1 comprised four acceleration profiles, with peak longitudinal accelerations of 0.2–0.3 g and nominal ramp jerk magnitudes of 0.5–1.5 g/s. Experiment 2 comprised nine regenerative-braking profiles, with target longitudinal acceleration levels of − 0.10, − 0.15, and − 0.20 g and time-to-target values of 0.3–2.1 s. The corresponding nominal ramp jerk magnitudes ranged from 0.071 to 0.667 g/s. Each of the 13 profiles was administered in a separate 30-min driving session on a different day. Thus, each participant was scheduled to complete four acceleration sessions and nine deceleration sessions in a within-subject design. The vehicle longitudinal control system was configured to track the predefined target acceleration or deceleration for each profile.

### Statistical analysis

Four MISC-derived indices were calculated for each 30-min driving session. MISCmean was defined as the mean of the 15 ratings obtained during the session, MISCmax as the maximum rating, MISCcumulative as the sum of the ratings, and MISClast as the final rating obtained at 30 min. For each participant, these session-level indices were then averaged across the four acceleration-profile sessions or the nine deceleration-profile sessions to obtain participant-level outcomes for Experiments 1 and 2, respectively. The analyses focused on inter-individual differences under each experimental condition rather than profile-specific effects. Participants with a participant-level MISCmax of 2 or higher were included in the symptomatic subgroup.

The reliability of the MISC-derived indices across repeated driving sessions was assessed separately for the acceleration and deceleration conditions using two-way mixed-effects intraclass correlation coefficients (ICCs) with absolute agreement. Participants were treated as random effects, and motion profiles were treated as fixed effects. Single-session ICCs [ICC(A,1)] and average-measure ICCs based on four acceleration sessions [ICC(A,4)] or nine deceleration sessions [ICC(A,9)] were calculated. Because the correlation analyses used participant-level outcomes averaged across sessions, the average-measure ICCs were used to evaluate the reliability of these values. 95% confidence intervals were estimated using 10,000 participant-level bootstrap samples.

Pearson correlation coefficients were calculated to examine associations among the MSSQ, VTC, and participant-level MISC-derived outcomes. Correlations were calculated for the full sample and the symptomatic subgroup in each experiment. The association between the VTC and MISCmax in the symptomatic subgroup under the deceleration condition was designated as the primary analysis on theoretical grounds. Because this was a single primary test, no adjustment for multiple comparisons was applied. The associations of the VTC with MISCmean, MISCcumulative, and MISClast in the same subgroup and condition were treated as supporting analyses, and their p values were adjusted using the Benjamini–Hochberg false-discovery-rate procedure^45^. All other correlations were treated as exploratory and are reported without adjustment, with emphasis on effect sizes and the direction of association.

Receiver operating characteristic (ROC) analyses were conducted at the participant level to compare the discriminative ability of the MSSQ and the VTC. For each experiment and threshold *k*, a participant was classified as positive if the highest MISC rating observed across that participant’s driving sessions was greater than or equal to k. The MSSQ and VTC were evaluated separately as continuous predictors, with higher values specified as indicating greater risk. Thresholds from *k* = 1 to *k* = 8 were examined as a sensitivity analysis, and the nausea threshold (MISC ≥ 6) was used as the primary reference. Areas under the ROC curves (AUCs) for the MSSQ and VTC were compared using DeLong’s test for two correlated ROC curves. 95% confidence intervals and the numbers of positive and negative participants were calculated for each threshold.

Analyses were performed using Python 3.13.5. All statistical tests were two-sided, and *p* < 0.05 was considered statistically significant.

## Experimental results

**Fig. 5:**
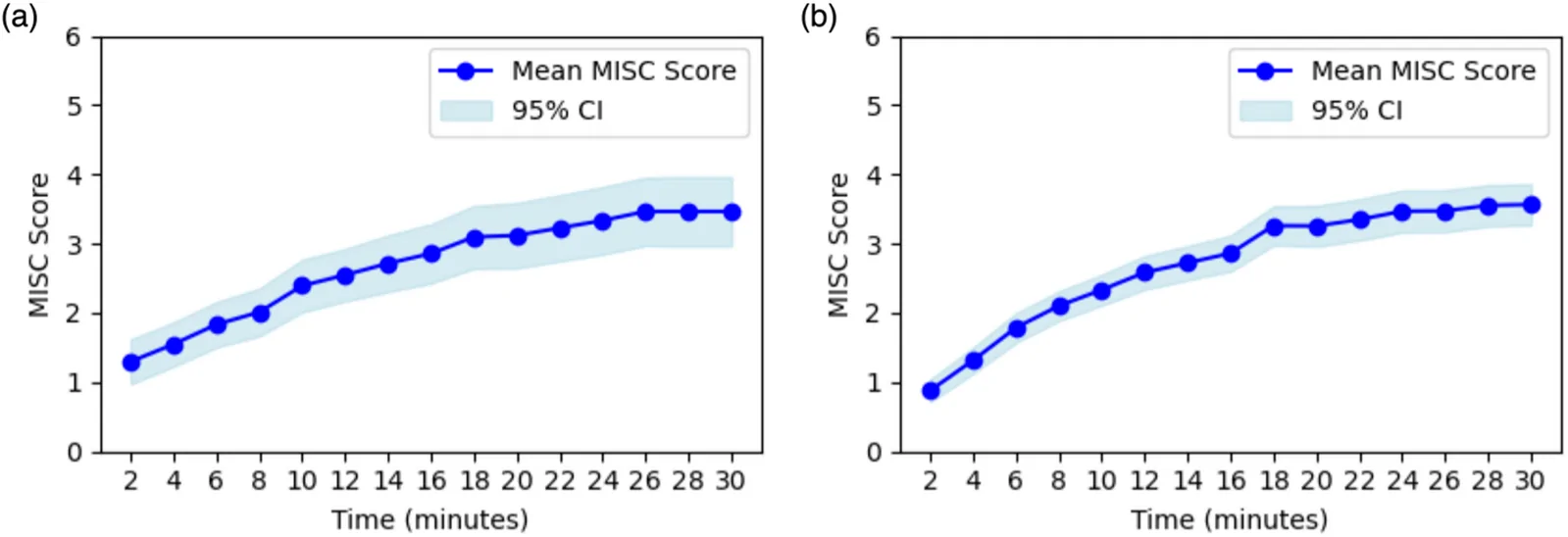
Temporal changes in MISC ratings during the 30-min driving sessions under the (**a**) acceleration and (**b**) deceleration conditions. MISC, Misery Scale.

Figure 5 shows the temporal changes in MISC ratings during the 30-min driving sessions under the acceleration and deceleration conditions. For each participant, ratings at each time point were averaged across the four acceleration-profile sessions or nine deceleration-profile sessions. The figure presents the mean participant-level ratings and their 95% confidence intervals. Under both conditions, mean MISC ratings increased progressively during the first 18 min and then showed relatively little further change. Mean ratings increased from values close to zero at 2 min to approximately 3.0 at 18 min and reached approximately 3.4 under the acceleration condition and 3.7 under the deceleration condition at 30 min. Descriptively, ratings increased somewhat more rapidly under the deceleration condition between 4 and 10 min and remained slightly higher toward the end of the exposure. The following analyses examine the associations of the MSSQ and VTC with participant-level MISC-derived outcomes.

### Relationship between MSSQ and vestibular time constant

**Fig. 6:**
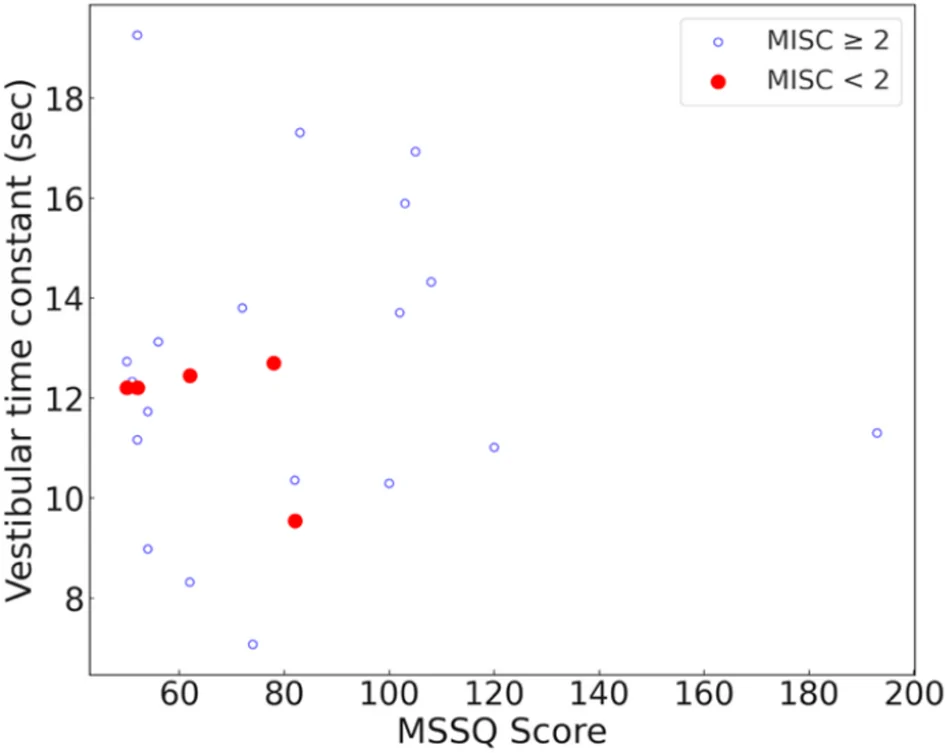
Relationship between MSSQ scores and vestibular time constants across participants. MSSQ, Motion Sickness Susceptibility Questionnaire; VTC, vestibular time constant.

Figure 6 shows the relationship between MSSQ scores and VTC values across the 24 participants. Pearson correlation analysis showed no significant linear association between the two measures (*r* = 0.04, *p* = 0.852). A similar result was observed after excluding participants with minimal symptoms (MISCmax < 2), with the correlation remaining nonsignificant (*r* = 0.06, *p* > 0.05, *N* = 19). Although no significant linear association was identified, exploratory inspection suggested a possible grouping pattern. Participants with minimal symptoms (MISCmax < 2) tended to cluster at lower VTC values ($\:\le\:$ 12.7 s) and MSSQ scores between 50 and 82, whereas participants who developed symptoms included most of those with VTC values $\:\ge\:$ 13 s and MSSQ scores $\:\ge\:$83. These values represent descriptive patterns observed in this sample rather than prespecified or validated cut-offs. The individual-level values underlying this observation are provided in Supplementary Table S1.

### Reliability of motion sickness measures

**Fig. 7:**
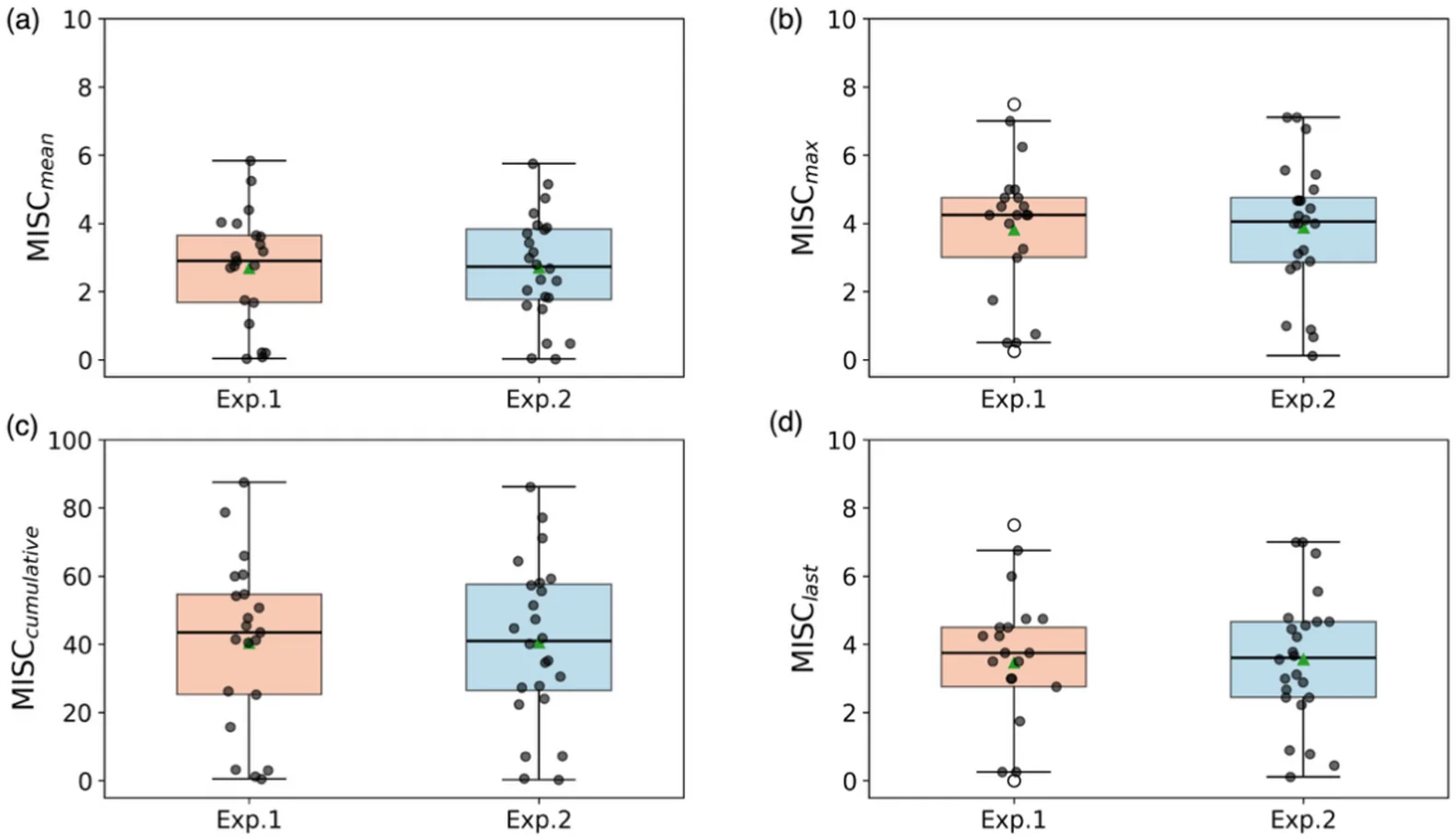
Distributions of the MISC-derived indices under the acceleration (Experiment 1) and deceleration (Experiment 2) conditions: (**a**) MISCmean, (**b**) MISCmax, (**c**) MISCcumulative, and (**d**) MISClast. MISC, Misery Scale.

Figure 7 shows the distributions of the participant-level MISC-derived indices averaged across the repeated motion-profile sessions in each experimental condition. Single-session ICCs ranged from 0.733 to 0.824 under the acceleration condition and from 0.711 to 0.787 under the deceleration condition. The session-averaged indices used in the participant-level analyses had higher ICCs. Under the acceleration condition, ICC(A,4) values were 0.949 (95% CI, 0.871–0.979) for MISCmean, 0.934 (95% CI, 0.826–0.971) for MISCmax, 0.949 (95% CI, 0.871–0.979) for MISCcumulative, and 0.917 (95% CI, 0.769–0.966) for MISClast. Under the deceleration condition, ICC(A,9) values were 0.971 (95% CI, 0.936–0.985), 0.959 (95% CI, 0.906–0.978), 0.971 (95% CI, 0.936–0.985), and 0.957 (95% CI, 0.907–0.976), respectively. These results support the use of session-averaged MISC-derived indices as participant-level outcomes in the subsequent analyses.

### Associations between MSSQ scores and motion sickness measures

#### Acceleration condition (experiment 1)

**Fig. 8:**
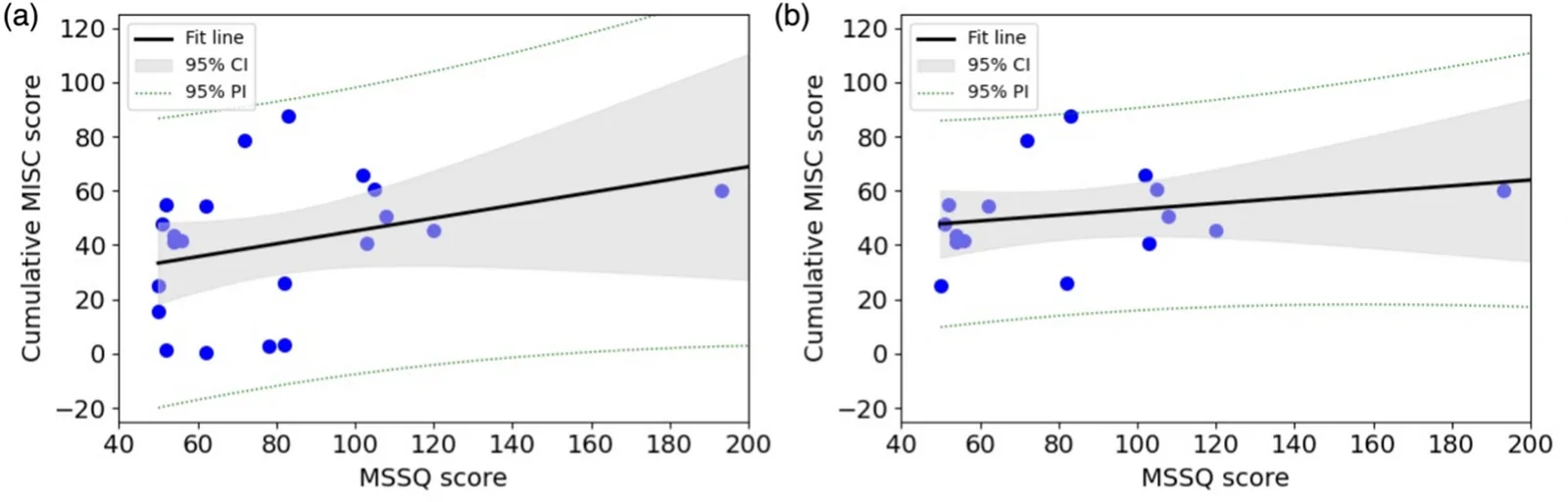
Relationship between MSSQ scores and MISCcumulative under the acceleration condition in the (**a**) full sample (*N* = 21) and (**b**) symptomatic subgroup (*N* = 16). MSSQ, Motion Sickness Susceptibility Questionnaire; MISC, Misery Scale.

The Experiment 1 analyses were based on data from 21 participants. The correlations between MSSQ scores and the participant-level MISC-derived outcomes were positive but not statistically significant. The correlation coefficients were *r* = 0.32 for MISCmean, *r* = 0.28 for MISCmax, *r* = 0.32 for MISCcumulative, and *r* = 0.29 for MISClast (*p* = 0.153–0.227). In the symptomatic subgroup, defined as participants with MISCmax ≥ 2 (*N* = 16), the corresponding coefficients were *r* = 0.24, 0.16, 0.24, and 0.20, respectively (*p* = 0.364–0.560). Table 1 presents the complete correlation results. Figure 8 shows the relationship between MSSQ scores and MISCcumulative in the full sample and symptomatic subgroup. Each point represents a participant-level MISCcumulative value averaged across the four acceleration-profile sessions. Solid lines indicate the fitted linear relationships, shaded areas indicate 95% confidence intervals, and dashed lines indicate 95% prediction intervals. The corresponding scatterplots for the other MISC-derived outcomes are provided in Supplementary Figures S1 and S2.

**Table 1:** Pearson correlation coefficients (*r*) between MSSQ scores and MISC-derived outcomes under the acceleration condition in the full sample (*N* = 21) and symptomatic subgroup (*N* = 16).

Group	MISCmean	MISCmax	MISCcumulative	MISClast
*N* = 21	0.32	0.28	0.32	0.29
*N* = 16	0.24	0.16	0.24	0.20

MSSQ, Motion Sickness Susceptibility Questionnaire; MISC, Misery Scale.

#### Deceleration condition (experiment 2)

**Fig. 9:**
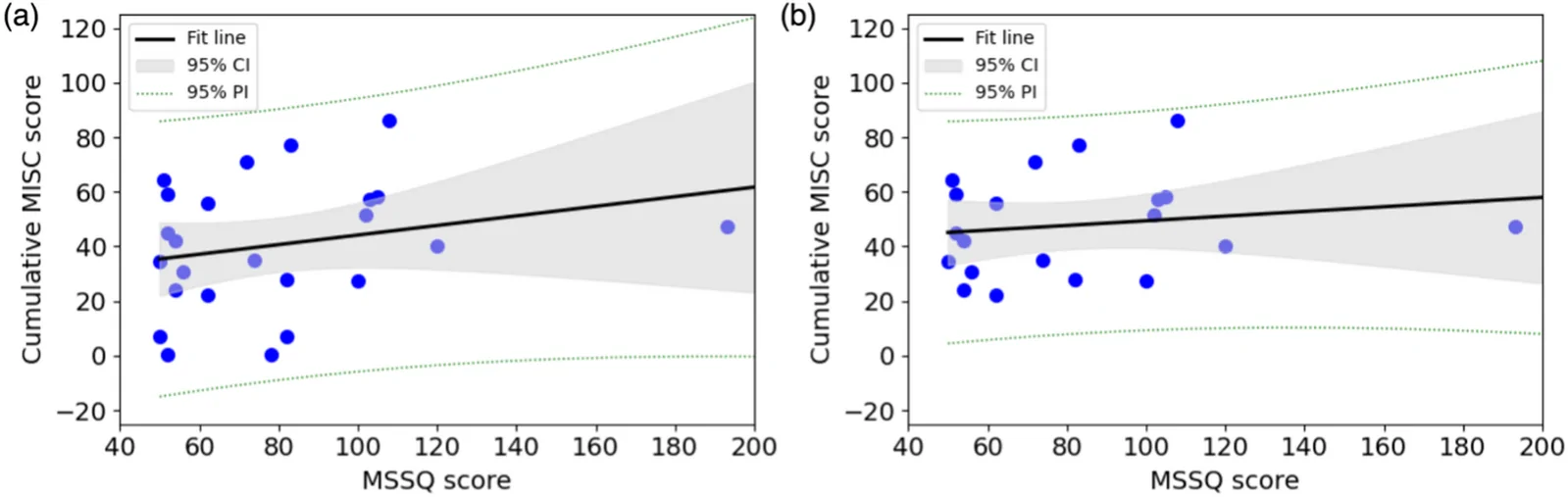
Relationship between MSSQ scores and MISCcumulative under the deceleration condition in the (**a**) full sample (*N* = 24) and (**b**) symptomatic subgroup (*N* = 20). MSSQ, Motion Sickness Susceptibility Questionnaire; MISC, Misery Scale.

All 24 participants completed Experiment 2. The correlations between MSSQ scores and the participant-level MISC-derived outcomes were positive but not statistically significant in the full sample. The correlation coefficients were *r* = 0.24 for MISCmean, *r* = 0.18 for MISCmax, *r* = 0.24 for MISCcumulative, and *r* = 0.23 for MISClast (*p* = 0.250–0.402). In the symptomatic subgroup (MISCmax ≥ 2; *N* = 20), the corresponding coefficients were *r* = 0.16, 0.06, 0.16, and 0.14, respectively (*p* = 0.491–0.794). Table 2 presents the complete correlation results. Figure 9 shows the relationship between MSSQ scores and MISCcumulative in the full sample and symptomatic subgroup. Each point represents a participant-level MISCcumulative value averaged across the nine deceleration-profile sessions. Solid lines indicate the fitted linear relationships, shaded areas indicate 95% confidence intervals, and dashed lines indicate 95% prediction intervals. The corresponding scatterplots for the other MISC-derived outcomes are provided in Supplementary Figures S3 and S4.

**Table 2:** Pearson correlation coefficients (r) between MSSQ scores and MISC-derived outcomes under the deceleration condition in the full sample (*N* = 24) and symptomatic subgroup (*N* = 20).

Group	MISCmean	MISCmax	MISCcumulative	MISClast
*N* = 24	0.24	0.18	0.24	0.23
*N* = 20	0.16	0.06	0.16	0.14

MSSQ, Motion Sickness Susceptibility Questionnaire; MISC, Misery Scale.

### Associations between VTC and motion sickness measures

#### Acceleration condition (experiment 1)

**Fig. 10:**
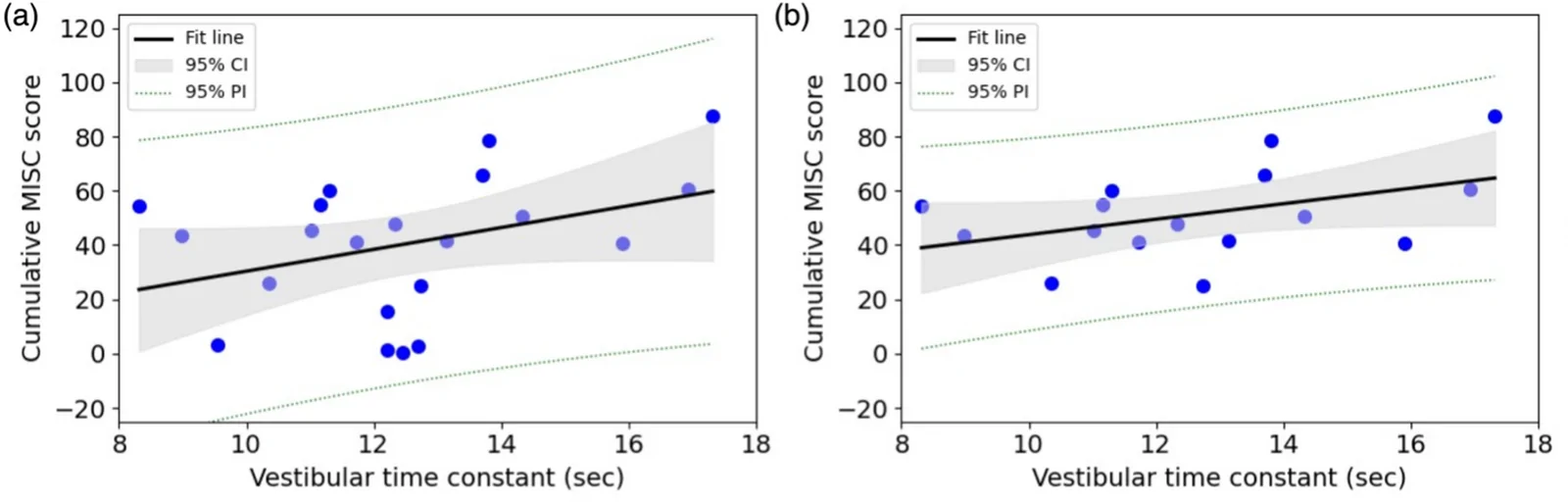
Relationship between VTC and MISCcumulative under the acceleration condition in the (**a**) full sample (*N* = 21) and (**b**) symptomatic subgroup (*N* = 16). VTC, vestibular time constant; MISC, Misery Scale.

In the full sample (*N* = 21), VTC showed consistently positive correlations with the four participant-level MISC-derived outcomes. The correlation coefficients and unadjusted *p* values were *r* = 0.38 and *p* = 0.093 for MISCmean, *r* = 0.37 and *p* = 0.098 for MISCmax, *r* = 0.38 and *p* = 0.093 for MISCcumulative, and *r* = 0.40 and *p* = 0.071 for MISClast. Thus, none of these exploratory associations reached the prespecified significance threshold of *p* < 0.05, although all *p* values were below 0.10. In the symptomatic subgroup (MISCmax $\:\ge\:$ 2; *N* = 16), the corresponding results were *r* = 0.44 and *p* = 0.085 for MISCmean, *r* = 0.47 and *p* = 0.066 for MISCmax, *r* = 0.44 and *p* = 0.085 for MISCcumulative, and *r* = 0.51 and *p* = 0.045 for MISClast. These Experiment 1 analyses were exploratory, and their *p* values were not adjusted for multiple comparisons. Table 3 presents the correlation coefficients. Figure 10 shows the relationship between VTC and MISCcumulative in the full sample and symptomatic subgroup. Each point represents a participant-level MISCcumulative value averaged across the four acceleration-profile sessions. Solid lines indicate the fitted linear relationships, shaded areas indicate 95% confidence intervals, and dashed lines indicate 95% prediction intervals. The corresponding scatterplots for the other MISC-derived outcomes are provided in Supplementary Figures S5 and S6.

**Table 3:** Pearson correlation coefficients (*r*) between VTC and MISC-derived outcomes under the acceleration condition in the full sample (*N* = 21) and symptomatic subgroup (*N* = 16).

Group	MISCmean	MISCmax	MISCcumulative	MISClast
*N* = 21	0.38	0.37	0.38	0.40
*N* = 16	0.44	0.47	0.44	0.51

VTC, vestibular time constant; MISC, Misery Scale.

#### Deceleration condition (experiment 2)

**Fig. 11:**
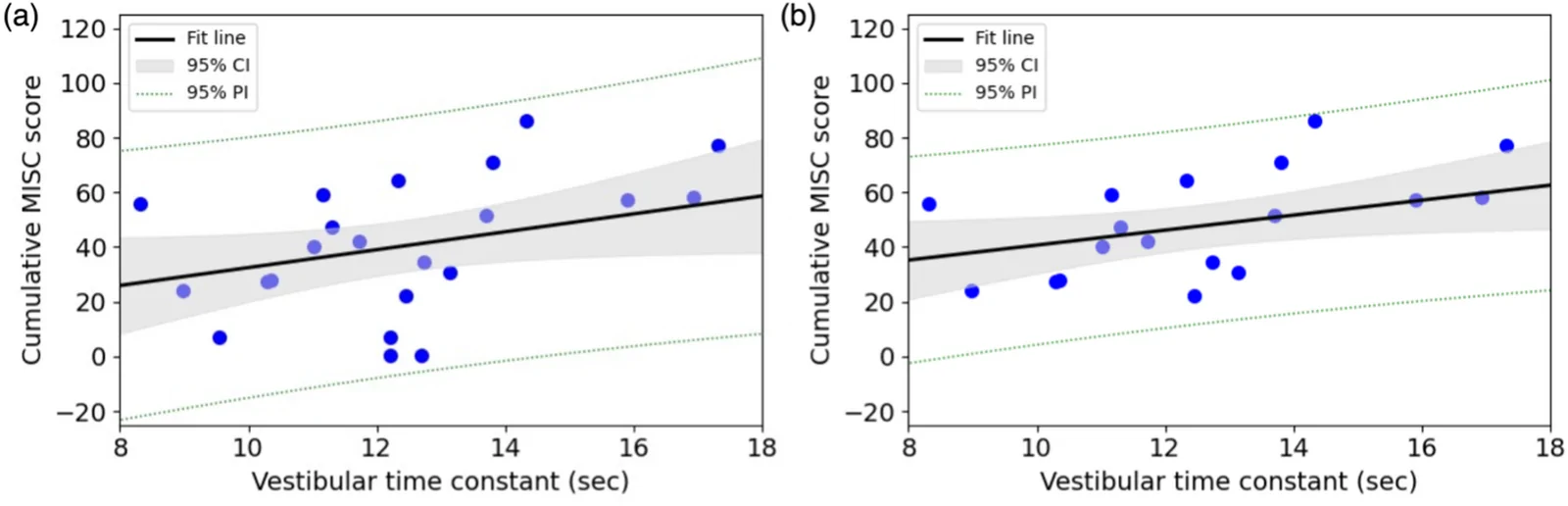
Relationship between VTC and MISCcumulative under the deceleration condition in the (**a**) full sample (*N* = 24) and (**b**) symptomatic subgroup (*N* = 20). VTC, vestibular time constant; MISC, Misery Scale.

In the full sample (*N* = 24), VTC was positively correlated with all four participant-level MISC-derived outcomes. The correlation coefficients were *r* = 0.40 for MISCmean, *r* = 0.45 for MISCmax, *r* = 0.40 for MISCcumulative, and *r* = 0.46 for MISClast. The associations with MISCmax (*p* = 0.027) and MISClast (*p* = 0.023) were statistically significant, whereas those with MISCmean and MISCcumulative did not reach the *p* < 0.05 threshold (both *p* = 0.053). In the symptomatic subgroup (MISCmax $\:\ge\:$ 2; *N* = 20), the primary association between VTC and MISCmax was *r* = 0.58 (*p* = 0.0069). The supporting associations were *r* = 0.46 for MISCmean, *r* = 0.46 for MISCcumulative, and *r* = 0.56 for MISClast. All three supporting associations remained statistically significant after Benjamini–Hochberg correction (MISCmean, *q* = 0.040; MISCcumulative, *q* = 0.040; MISClast, *q* = 0.032). Figure 11 shows the relationship between VTC and MISCcumulative in the full sample and symptomatic subgroup. Each point represents a participant-level MISCcumulative value averaged across the nine deceleration-profile sessions. Solid lines indicate the fitted linear relationships, shaded areas indicate 95% confidence intervals, and dashed lines indicate 95% prediction intervals. The corresponding scatterplots for the other MISC-derived outcomes are provided in Supplementary Figures S7 and S8. Table 4 presents the correlation coefficient.

**Table 4:** Pearson correlation coefficients (*r*) between VTC and MISC-derived outcomes under the deceleration condition in the full sample (*N* = 24) and symptomatic subgroup (*N* = 20).

Group	MISCmean	MISCmax	MISCcumulative	MISClast
*N* = 24	0.40	0.45	0.40	0.46
*N* = 20	0.46	0.58	0.46	0.56

VTC, vestibular time constant; MISC, Misery Scale.

### Comparison of MSSQ and VTC correlations with motion sickness metrics

**Fig. 12:**
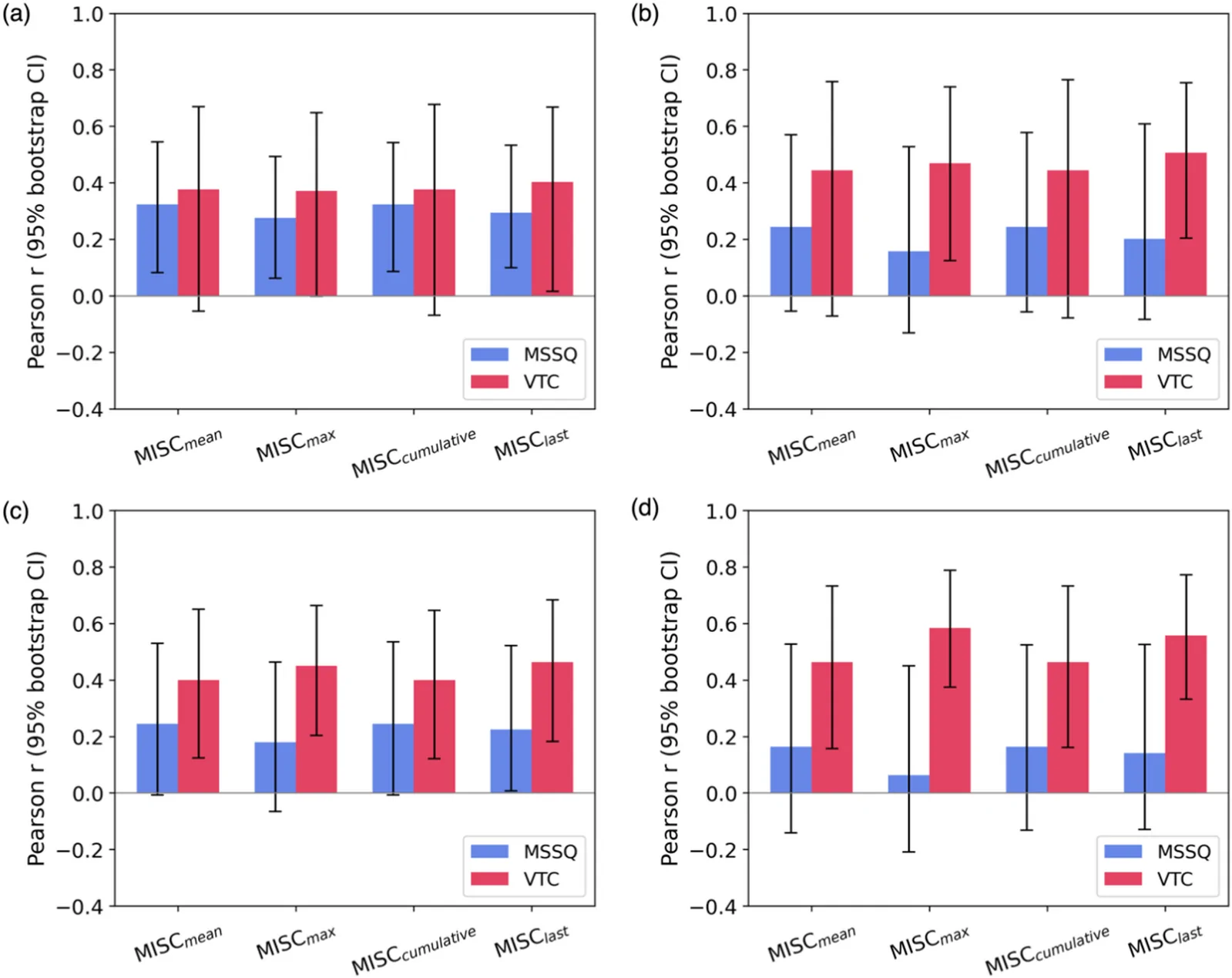
Correlations of the MSSQ and VTC with the MISC-derived outcomes. (**a**) Experiment 1, full sample (*N* = 21); (**b**) Experiment 1, symptomatic subgroup (*N* = 16); (**c**) Experiment 2, full sample (*N* = 24); and (**d**) Experiment 2, symptomatic subgroup (*N* = 20). MSSQ, Motion Sickness Susceptibility Questionnaire; VTC, vestibular time constant; MISC, Misery Scale.

Figure 12 compares the Pearson correlation coefficients of the MSSQ and VTC with the four participant-level MISC-derived outcomes. Bars represent the correlation coefficients, and error bars show participant-level bootstrap 95% confidence intervals. Under the acceleration condition, MSSQ correlation coefficients ranged from *r* = 0.28 to 0.32 in the full sample and from *r* = 0.16 to 0.24 in the symptomatic subgroup. The corresponding VTC coefficients ranged from *r* = 0.37 to 0.40 and from *r* = 0.44 to 0.51, respectively. Under the deceleration condition, MSSQ correlation coefficients ranged from *r* = 0.18 to 0.24 in the full sample and from *r* = 0.06 to 0.16 in the symptomatic subgroup. The corresponding VTC coefficients ranged from *r* = 0.40 to 0.46 and from *r* = 0.46 to 0.58, respectively. Across both experimental conditions and participant groups, the VTC showed consistently larger positive correlations with the MISC-derived outcomes than the MSSQ, with the largest differences observed in the symptomatic subgroup under the deceleration condition.

### Discriminative ability of the MSSQ and VTC

**Fig. 13:**
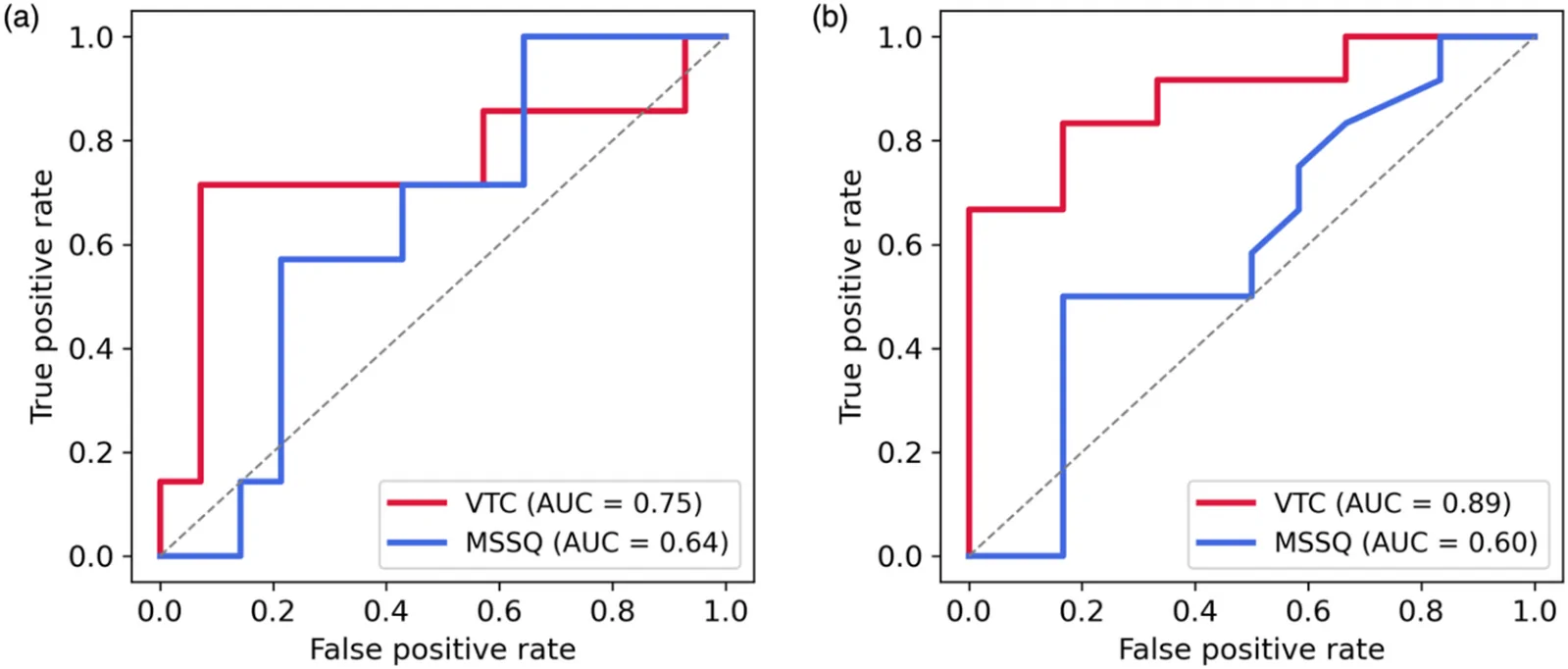
Participant-level ROC curves for the MSSQ and VTC at the nausea threshold (maximum MISC ≥ 6) under (**a**) acceleration and (**b**) deceleration. ROC, receiver operating characteristic; AUC, area under the curve; MSSQ, Motion Sickness Susceptibility Questionnaire; VTC, vestibular time constant; MISC, Misery Scale.

**Fig. 14:**
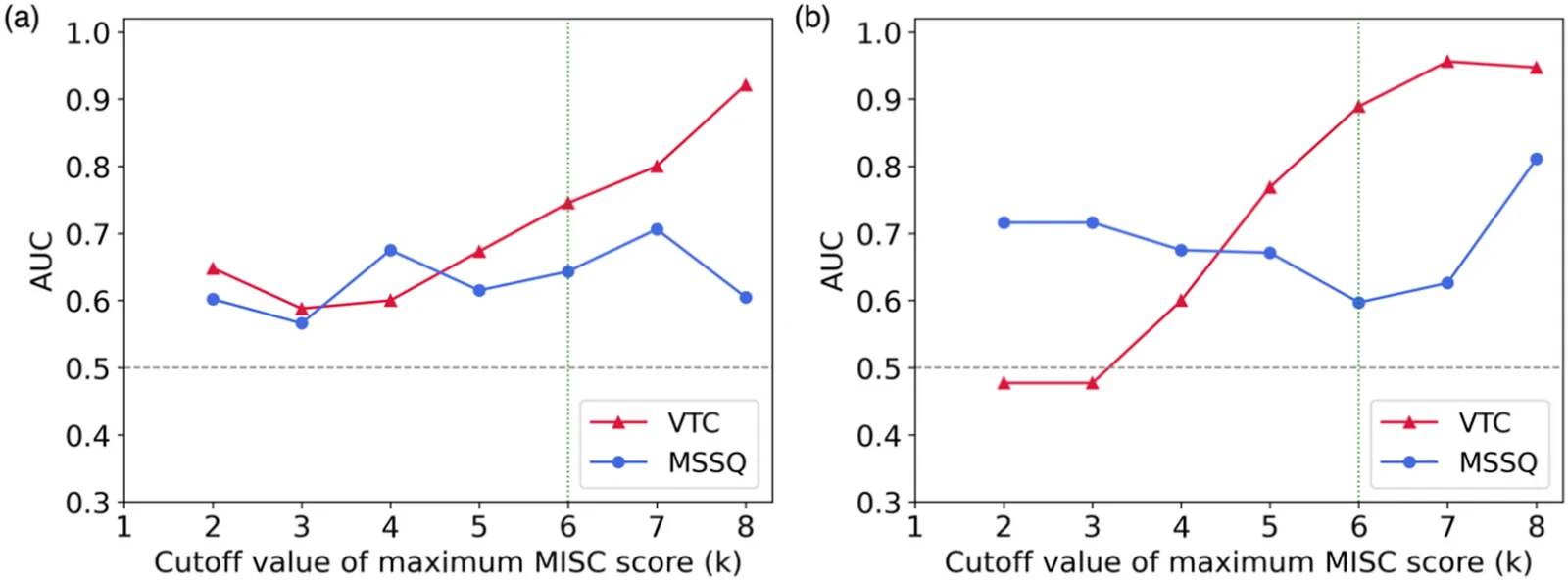
Participant-level AUC values for the MSSQ and VTC across maximum MISC thresholds under (**a**) acceleration and (**b**) deceleration. The dotted line marks the nausea threshold (MISC ≥ 6). AUC, area under the curve; MSSQ, Motion Sickness Susceptibility Questionnaire; VTC, vestibular time constant; MISC, Misery Scale.

Participant-level ROC analyses were conducted to compare the ability of the MSSQ and the VTC to discriminate participants who reached specified MISC thresholds. For each experiment and threshold *k*, participants were classified as positive if their highest MISC rating was greater than or equal to *k*. Thresholds from *k* = 1 to *k* = 8 were examined as a sensitivity analysis, with the nausea threshold (MISC ≥ 6) used as the primary reference. The AUCs were compared using DeLong’s test for correlated ROC curves. Table 5 presents the AUC values, and the corresponding 95% confidence intervals, DeLong *p* values, and numbers of positive and negative participants are provided in Supplementary Table S2. Figure 13 presents the ROC curves at the nausea threshold, Fig. 14 summarizes the AUC values across thresholds, and the complete set of ROC curves is provided in Supplementary Figures S9 and S10.

Under deceleration (Experiment 2), the VTC showed greater discriminative ability than the MSSQ at the nausea threshold (AUC 0.89, 95% CI 0.76–1.00, versus 0.60, 95% CI 0.36–0.84; DeLong *p* = 0.048). A similar difference was observed at *k* = 7 (AUC 0.96 versus 0.63; DeLong *p* = 0.007). Under acceleration (Experiment 1), the corresponding AUCs were 0.75 for the VTC and 0.64 for the MSSQ, with no significant difference between them (DeLong *p* = 0.423). Estimates at thresholds with very small positive or negative classes were not emphasized. These findings indicate greater discriminative ability of the VTC for nausea-level symptoms, particularly under deceleration.

**Table 5:** Participant-level AUC values for the MSSQ and VTC across maximum MISC thresholds in Experiments 1 and 2.

Maximum MISC threshold (k)	Experiment 1		Experiment 2	
	MSSQ AUC	VTC AUC	MSSQ AUC	VTC AUC
1	n.e.	n.e.	n.e.	n.e.
2	0.60	0.65	0.72	0.48
3	0.57	0.59	0.72	0.48
4	0.68	0.60	0.68	0.60
5	0.62	0.67	0.67	0.77
6	0.64	0.75	0.60	0.89
7	0.71	0.80	0.63	0.96
8	0.61	0.92	0.81	0.95

n.e., not estimable. AUC, area under the curve; MSSQ, Motion Sickness Susceptibility Questionnaire; VTC, vestibular time constant; MISC, Misery Scale.

## Discussion

### Interpretation of the findings

The high intraclass correlation coefficients across the repeated acceleration and deceleration profiles indicate that inter-individual differences in the MISC-derived outcomes were largely consistent across sessions. This consistency supports the use of outcomes averaged across profiles to characterize participant-level motion sickness susceptibility. Together with the observed associations between the VTC and symptom severity, these findings support the consideration of the VTC as a physiological marker of individual susceptibility.

The VTC showed positive associations with motion sickness severity during controlled on-road driving. These associations were strongest and most consistent under deceleration, particularly in the symptomatic subgroup, whereas the results under acceleration were weaker and less consistent. The strongest association was observed between the VTC and peak symptom severity (MISCmax) in the symptomatic subgroup under deceleration. These findings extend previous evidence linking the VTC to seasickness susceptibility^33^ to an automotive setting involving longitudinal and lateral vehicle motion.

The participant-level ROC analyses provided convergent evidence regarding the discriminative ability of the VTC. At the nausea threshold, the VTC showed a higher AUC than the MSSQ under both driving conditions, with a significant difference under deceleration. The VTC–MSSQ difference was also significant at *k* = 7 under deceleration. At lower thresholds, the small number of negative cases precluded a clear comparison of the two measures. Taken together, the correlation and ROC results indicate that the VTC was more informative than the MSSQ for nausea-level symptoms, particularly under deceleration. The MSSQ nevertheless remains an accessible measure of self-reported motion sickness history^46^, while the VTC provides objective physiological information not captured by the questionnaire.

The more consistent VTC associations observed under deceleration may reflect differences in the direction and temporal characteristics of the motion stimuli. Nominal ramp jerk magnitudes were 0.071–0.667 g/s in Experiment 2 and 0.5–1.5 g/s in Experiment 1, indicating that the stronger findings under deceleration were not associated with greater longitudinal jerk magnitude. Instead, braking-specific temporal characteristics and the direction of longitudinal acceleration may produce vestibular–visual dynamics that interact with individual differences in vestibular signal retention. In individuals with longer VTCs, prolonged vestibular signals may increase mismatch with visual and proprioceptive inputs during braking. This interpretation is consistent with sensory-conflict and velocity-storage models of motion sickness^47–49^, although studies that independently manipulate acceleration direction and temporal profile are needed to identify the responsible stimulus characteristics.

Overall, this study extends evidence for the association between the VTC and motion sickness susceptibility to controlled on-road driving. Participant-level MISC-derived outcomes were reliable across repeated motion profiles, and longer VTCs were associated with greater symptom severity, particularly under deceleration and among symptomatic participants. At the nausea threshold, the VTC showed greater discriminative ability than the MSSQ under deceleration. These convergent findings support the potential value of the VTC as a complementary physiological marker for motion sickness susceptibility assessment in automotive settings.

### Limitations and future directions

This study involved a relatively small sample of female adults. Although testing sessions were scheduled outside menstruation, cycle phase was not otherwise controlled. Because the study did not include male participants or establish a validated clinical VTC cut-off, the present data cannot determine whether VTC distributions or discriminative thresholds would differ in a gender-inclusive population. Future studies with larger samples including both female and male participants should examine the generalizability of the VTC–symptom association and potential differences in VTC distributions and candidate cut-offs related to sex and menstrual cycle phase. Motion sickness severity was assessed using the self-reported MISC. Physiological measures such as heart rate variability, electroencephalography, or other biosignals could complement these symptom ratings in future studies^50^. EOG was used to estimate the VTC from horizontal slow-phase eye velocity during the short rotary-chair examination. Future studies could additionally use video-oculography to permit direct visual inspection of eye movements and recording artifacts. SVV and SVH were also collected during the vestibular assessment but were outside the scope of the present analysis, which focused on the VTC. As measures of otolith function and visuo-vestibular integration^51,52^, their associations with motion sickness susceptibility could be examined in future analyses.

### Practical implications

The present findings suggest a potential role for the VTC in passenger-specific motion sickness mitigation in future mobility systems. Information on vestibular susceptibility could inform trajectory planning and the design of acceleration, deceleration, and jerk profiles to improve passenger comfort. The MSSQ could provide an accessible assessment of previous motion sickness experience, while the VTC could add objective information on vestibular processing. These measures could also be incorporated into adaptive vehicle-control strategies alongside visual, auditory, or haptic anticipatory cues^12,15,53,54^. The observed discriminative ability of the VTC, particularly under deceleration, supports further development of passenger-specific motion sickness assessment and mitigation strategies.

## Conclusion

This study examined the associations of the MSSQ and VTC with motion sickness severity under controlled on-road acceleration and deceleration conditions. The VTC was positively associated with symptom severity, with the strongest and most consistent associations observed under deceleration and among participants who developed symptoms, whereas the MSSQ showed weaker, largely non-significant associations. Participant-level ROC analyses further indicated that the VTC discriminated participants who reached the nausea threshold better than the MSSQ, with a significant difference under deceleration. These findings extend evidence for the relevance of the VTC to automotive motion sickness and indicate that it provides objective physiological information not captured by the MSSQ. The VTC may therefore complement questionnaire-based susceptibility assessment and support the development of passenger-specific motion sickness mitigation strategies in future mobility systems.

## Supplementary Information

Below is the link to the electronic supplementary material.

Supplementary Material 1

## Data Availability

All data supporting the findings of this study are included in the article and its supplementary materials.

## Code availability

The analysis code reported in this study is openly available at Zenodo (https://doi.org/10.5281/zenodo.21791198).

## References

[1] Diels, C. & Bos, J. E. Self-driving carsickness. *Appl. Ergon.* **53**, 374–382 (2016). 26446454 10.1016/j.apergo.2015.09.009

[2] Oman, C. M. A heuristic mathematical model for the dynamics of sensory conflict and motion sickness. *Acta Otolaryngol.* **94**, 4–44 (1982). 6303041

[3] Oman, C. M. Motion sickness: a synthesis and evaluation of the sensory conflict theory. *Can. J. Physiol. Pharmacol.* **68**, 294–303 (1990). 2178753 10.1139/y90-044

[4] Iskander, J. et al. From car sickness to autonomous car sickness: A review. *Transp. Res. F Traffic Psychol. Behav.* **62**, 716–726 (2019).

[5] Keshavarz, B. & Golding, J. F. Motion sickness: current concepts and management. *Curr. Opin. Neurol.* **35**, 107–112 (2022). 34839340 10.1097/WCO.0000000000001018

[6] Sivak, M. & Schoettle, B. *Motion Sickness in Self-Driving Vehicles* (University of Michigan, 2015).

[7] Lagami, D., Shupak, A., Jamison, A. & Tal, D. The vestibular time constant and clinical response to antimotion sickness medication. *Ear Hear.* **44**, 1404–1409 (2023). 37221635 10.1097/AUD.0000000000001385

[8] Molefi, E., McLoughlin, I. & Palaniappan, R. Transcutaneous auricular vagus nerve stimulation for visually induced motion sickness: An eloreta study. *Brain Topogr.* **38**, 11 (2025). 10.1007/s10548-024-01088-6 PMC11531436 39487878

[9] Molefi, E., McLoughlin, I. & Palaniappan, R. Symmetric projection attractor reconstruction: Transcutaneous auricular vagus nerve stimulation for visually induced motion sickness. *Auton. Neurosci.* **261**, 103318 (2025). 40683135 10.1016/j.autneu.2025.103318

[10] Griffin, M. J. & Newman, M. M. Visual field effects on motion sickness in cars. *Aviat. Space Environ. Med.* **75**, 739–748 (2004). 15460624

[11] Kuiper, O. X., Bos, J. E. & Diels, C. Looking forward: In-vehicle auxiliary display positioning affects carsickness. *Appl. Ergon.* **68**, 169–175 (2018). 29409631 10.1016/j.apergo.2017.11.002

[12] Kuiper, O. X., Bos, J. E., Diels, C. & Schmidt, E. A. Knowing what’s coming: Anticipatory audio cues can mitigate motion sickness. *Appl. Ergon.* **85**, 103068 (2020). 32174356 10.1016/j.apergo.2020.103068

[13] Li, D. & Chen, L. Mitigating motion sickness in automated vehicles with vibration cue system. *Ergonomics* **65**, 1313–1325 (2022). 35020579 10.1080/00140139.2022.2028902

[14] Reuten, A. et al. The (in) effectiveness of anticipatory vibrotactile cues in mitigating motion sickness. *Exp. Brain Res.* **241**, 1251–1261 (2023). 36971821 10.1007/s00221-023-06596-8 PMC10042112

[15] Emond, W., Bohrmann, D. & Zare, M. Will visual cues help alleviating motion sickness in automated cars? A review article. *Ergonomics* **67**, 772–800 (2024). 37981841 10.1080/00140139.2023.2286187

[16] Golding, J. F. Motion sickness susceptibility questionnaire revised and its relationship to other forms of sickness. *Brain Res. Bull.* **47**, 507–516 (1998). 10052582 10.1016/s0361-9230(98)00091-4

[17] Siddiqi, M. R., Milani, S., Jazar, R. N. & Marzbani, H. Ergonomic path planning for autonomous vehicles-an investigation on the effect of transition curves on motion sickness. *IEEE Trans. Intell. Transp. Syst.* **23**, 7258–7269 (2021).

[18] Htike, Z., Papaioannou, G., Siampis, E., Velenis, E. & Longo, S. Fundamentals of motion planning for mitigating motion sickness in automated vehicles. *IEEE Trans. Veh. Technol.* **71**, 2375–2384 (2021).

[19] Jain, V., Kumar, S. S., Papaioannou, G., Happee, R. & Shyrokau, B. Optimal trajectory planning for mitigated motion sickness: Simulator study assessment. *IEEE Trans. Intell. Transp. Syst.* **24**, 10653–10664 (2023).

[20] Golding, J. F., Bles, W., Bos, J., Haynes, T. & Gresty, M. A. Motion sickness and tilts of the inertial force environment: active suspension systems vs. active passengers. *Aviat. Space Environ. Med.* **74**, 220–227 (2003). 12650268

[21] Jones, M. L. et al. Motion sickness in passenger vehicles during test track operations. *Ergonomics* **62**, 1357–1371 (2019). 31282785 10.1080/00140139.2019.1632938

[22] Golding, J. F. Predicting individual differences in motion sickness susceptibility by questionnaire. *Pers. Indiv. Differ.* **41**, 237–248 (2006).

[23] Flanagan, M. B., May, J. G. & Dobie, T. G. Sex differences in tolerance to visually-induced motion sickness. *Aviat. Space Environ. Med.* **76**, 642–646 (2005). 16018346

[24] Lamb, S. & Kwok, K. C. MSSQ-short norms may underestimate highly susceptible individuals: updating the MSSQ-short norms. *Hum. Factors* **57**, 622–633 (2015). 25977322 10.1177/0018720814555862

[25] Raphan, T., Matsuo, V. & Cohen, B. Velocity storage in the vestibulo-ocular reflex arc (VOR). *Exp. Brain Res.* **35**, 229–248 (1979). 108122 10.1007/BF00236613

[26] Cohen, B., Dai, M. & Raphan, T. The critical role of velocity storage in production of motion sickness. *Ann. N. Y. Acad. Sci.* **1004**, 359–376 (2003). 14662476 10.1196/annals.1303.034

[27] Dai, M., Kunin, M., Raphan, T. & Cohen, B. The relation of motion sickness to the spatial–temporal properties of velocity storage. *Exp. Brain Res.* **151**, 173–189 (2003). 12783152 10.1007/s00221-003-1479-4

[28] Dai, M., Raphan, T. & Cohen, B. Spatial orientation of the vestibular system: dependence of optokinetic after-nystagmus on gravity. *J. Neurophysiol.* **66**, 1422–1439 (1991). 1761991 10.1152/jn.1991.66.4.1422

[29] Clement, G. & Reschke, M. F. Relationship between motion sickness susceptibility and vestibulo-ocular reflex gain and phase. *J. Vestib. Res.* **28**, 295–304 (2018). 29689763 10.3233/VES-180632

[30] Li, Y. et al. Quantitative study on objective indicators for assessing motion sickness susceptibility based on Vestibulo-Ocular Reflex experiments. *Sci. Rep.* **14**, 28782 (2024). 39567728 10.1038/s41598-024-80233-4 PMC11579332

[31] Dai, M., Raphan, T. & Cohen, B. Labyrinthine lesions and motion sickness susceptibility. *Exp. Brain Res.* **178**, 477–487 (2007). 17256169 10.1007/s00221-006-0759-1 PMC3181155

[32] Dai, M., Raphan, T. & Cohen, B. Prolonged reduction of motion sickness sensitivity by visual-vestibular interaction. *Exp. Brain Res.* **210**, 503–513 (2011). 21287155 10.1007/s00221-011-2548-8 PMC3182575

[33] Lagami, D., Gutkovich, Y. E., Jamison, A., Fonar, Y. & Tal, D. Seasickness susceptibility and the vestibular time constant: a prospective study. *Exp. Brain Res.* **242**, 267–274 (2024). 38015244 10.1007/s00221-023-06745-z

[34] Talsma, T. M. W., Hassanain, O., Happee, R. & de Winkel, K. N. Validation of a moving base driving simulator for motion sickness research. *Appl. Ergon.* **106**, 103897 (2023). 36206673 10.1016/j.apergo.2022.103897

[35] Bos, J. E., MacKinnon, S. N. & Patterson, A. Motion sickness symptoms in a ship motion simulator: effects of inside, outside, and no view. *Aviat. Space Environ. Med.* **76**, 1111–1118 (2005). 16370260

[36] Lackner, J. R. Motion sickness: more than nausea and vomiting. *Exp. Brain Res.* **232**, 2493–2510 (2014). 24961738 10.1007/s00221-014-4008-8 PMC4112051

[37] Cohen, B., Dai, M., Yakushin, S. B. & Raphan, T. Baclofen, motion sickness susceptibility and the neural basis for velocity storage. *Prog. Brain Res.* **171**, 543–553 (2008). 18718351 10.1016/S0079-6123(08)00677-8 PMC12964195

[38] Gimmon, Y. & Schubert, M. C. Vestibular testing-rotary chair and dynamic visual acuity tests. *Vestib. Disord*. **82**, 39–46 (2019). 10.1159/000490270 30947187

[39] Arriaga, M. A. et al. Rotational chair (ROTO) instead of electronystagmography (ENG) as the primary vestibular test. *Otolaryngol. Head Neck Surg.* **133**, 329–333 (2005). 10.1016/j.otohns.2005.05.002 16143176

[40] Meiry, J. L. *The Vestibular System and Human Dynamic Space Orientation* (Massachusetts Institute of Technology, 1965).

[41] Galiana, H. L. A nystagmus strategy to linearize the vestibulo-ocular reflex. *IEEE Trans. Biomed. Eng.* **38**, 532–543 (1991). 1879842 10.1109/10.81578

[42] Propper, R. E., Bonato, F., Ward, L. & Sumner, K. Findings of an effect of gender, but not handedness, on self-reported motion sickness propensity. *Biopsychosoc. Med.* **12**, 1–4 (2018).29434655

[43] Schmid-Priscoveanu, A. & Allum, J. Die infrarot-und die videookulographie–alternativen zur Elektrookulographie? *HNO* **47**, 472–478 (1999). 10412656 10.1007/s001060050407

[44] Choi, H. G., Hong, S. K., Park, S. K., Lee, H. J. & Chang, J. Acute alcohol intake impairs the velocity storage mechanism and affects both high-frequency vestibular-ocular reflex and postural control. *Int. J. Environ. Res. Public Health* **19**, 3911 (2022). 35409596 10.3390/ijerph19073911 PMC8997842

[45] Benjamini, Y. & Hochberg, Y. Controlling the false discovery rate: a practical and powerful approach to multiple testing. *J. R. Stat. Soc. Ser. B (Methodol.)* **57**, 289–300 (1995).

[46] Tian, Z. S. et al. Evaluation of intelligent electric vehicle carsickness performance considering passenger motion sensitivity. *Proc. Inst. Mech. Eng. D J. Autom. Eng.* **239**, 7076–7089 (2025).

[47] Bertolini, G. & Straumann, D. Moving in a moving world: a review on vestibular motion sickness. *Front. Neurol.* **7**, 14 (2016). 26913019 10.3389/fneur.2016.00014 PMC4753518

[48] Madhani, A., Lewis, R. F. & Karmali, F. How peripheral vestibular damage affects velocity storage: a causative explanation. *J. Assoc. Res. Otolaryngol.* **23**, 551–566 (2022). 35768706 10.1007/s10162-022-00853-3 PMC9437187

[49] Waespe, W., Cohen, B. & Raphan, T. Role of the flocculus and paraflocculus in optokinetic nystagmus and visual-vestibular interactions: effects of lesions. *Exp. Brain Res.* **50**, 9–33 (1983). 6357831 10.1007/BF00238229

[50] Bang, J. S., Won, D. O., Kam, T. E. & Lee, S. W. Motion sickness prediction based on dry EEG in real driving environment. *IEEE Trans. Intell. Transp. Syst.* **24**, 5442–5455 (2023).

[51] Böhmer, A. & Mast, F. Assessing otolith function by the subjective visual vertical. *Ann. N. Y. Acad. Sci.* **871**, 221–231 (1999). 10372074 10.1111/j.1749-6632.1999.tb09187.x

[52] Dieterich, M. & Brandt, T. Ocular torsion and tilt of subjective visual vertical are sensitive brainstem signs. *Ann. Neurol.* **33**, 292–299 (1993). 8498813 10.1002/ana.410330311

[53] Reuten, A. J. C., Yunus, I., Bos, J. E., Martens, M. H. & Smeets, J. B. J. Anticipatory cues can mitigate car sickness on the road. *Transp. Res. F Traffic Psychol. Behav.* **105**, 196–205 (2024).

[54] Pereira, E. et al. Motion sickness countermeasures for autonomous driving: Trends and future directions. *Transp. Eng.* **15**, 100220 (2024).

