# Supplementary material for: Vestibular time constant and individual susceptibility to motion sickness in real-world driving
Source: Sci Rep. 2026 Sep 6;16:27829. doi: 10.1038/s41598-026-68887-8 (PMC13547338; doi:10.1038/s41598-026-68887-8)
Supplement: Supplementary file 1 [file 41598_2026_68887_MOESM1_ESM.docx]

**Supplementary Table S1.** Individual participant data for VTC measurements and MSSQ scores. Values include per- and post-rotary results for both rightward and leftward rotations, as well as mean VTC values. VTC, vestibular time constant; MSSQ, Motion Sickness Susceptibility Questionnaire.

| Subject No. | Age | Nystagmus test result (sec) | | | | Vestibular time constant (sec) | | | MSSQ  score |
| --- | --- | --- | --- | --- | --- | --- | --- | --- | --- |
|  |  | Right direction | | Left direction | |  |  |  |  |
|  |  | Per-Rotary | Post-Rotary | Per-Rotary | Post-Rotary | Right direction | Left direction | Mean |  |
| 1 | 34 | 18.40 | 9.89 | 11.62 | 15.30 | 16.85 | 10.76 | 13.80 | 72 |
| 2 | 29 | 13.03 | 14.64 | 10.54 | 11.60 | 12.32 | 12.59 | 12.45 | 62 |
| 3 | 39 | 12.61 | 13.29 | 10.01 | 8.75 | 10.68 | 11.65 | 11.17 | 52 |
| 4 | 26 | 13.24 | 13.46 | 11.67 | 12.43 | 12.84 | 12.57 | 12.70 | 78 |
| 5 | 32 | 13.06 | 11.59 | 8.22 | 8.56 | 10.81 | 9.91 | 10.36 | 82 |
| 6 | 38 | 8.88 | 10.04 | 12.95 | 16.97 | 12.93 | 11.50 | 12.21 | 52 |
| 7 | 36 | 11.37 | 15.37 | 12.49 | 9.60 | 10.49 | 13.93 | 12.21 | 50 |
| 8 | 23 | 15.96 | 15.34 | 11.43 | 9.77 | 12.87 | 13.39 | 13.13 | 56 |
| 9 | 40 | 20.63 | 17.62 | 11.16 | 19.84 | 20.24 | 14.39 | 17.31 | 83 |
| 10 | 38 | 10.12 | 8.50 | 13.80 | 11.63 | 10.88 | 11.15 | 11.01 | 120 |
| 11 | 28 | 17.48 | 8.34 | 6.63 | 14.47 | 15.98 | 7.49 | 11.73 | 54 |
| 12 | 40 | 18.32 | 10.13 | 15.85 | 19.28 | 18.80 | 12.99 | 15.90 | 103 |
| 13 | 29 | 12.49 | 11.08 | 8.77 | 12.86 | 12.68 | 9.93 | 11.30 | 193 |
| 14 | 40 | 10.45 | 16.32 | 12.45 | 11.71 | 11.08 | 14.39 | 12.73 | 50 |
| 15 | 26 | 22.71 | 14.38 | 11.77 | 18.87 | 20.79 | 13.08 | 16.93 | 105 |
| 16 | 30 | 13.44 | 17.32 | 10.57 | 13.50 | 13.47 | 13.95 | 13.71 | 102 |
| 17 | 36 | 18.18 | 12.04 | 13.28 | 13.79 | 15.99 | 12.66 | 14.32 | 108 |
| 18 | 26 | 11.65 | 7.84 | 5.08 | 8.70 | 10.18 | 6.46 | 8.32 | 62 |
| 19 | 40 | 10.39 | 12.62 | 9.91 | 16.41 | 13.40 | 11.27 | 12.33 | 51 |
| 20 | 27 | 6.49 | 8.60 | 8.60 | 12.23 | 9.36 | 8.60 | 8.98 | 54 |
| 21 | 33 | 8.33 | 6.06 | 7.30 | 6.60 | 7.47 | 6.68 | 7.07 | 74 |
| 22 | 36 | 17.37 | 22.78 | 17.31 | 19.59 | 18.48 | 20.05 | 19.26 | 52 |
| 23 | 35 | 11.40 | 9.93 | 10.06 | 9.78 | 10.59 | 10.00 | 10.29 | 100 |
| 24 | 36 | 12.11 | 10.67 | 8.15 | 7.24 | 9.68 | 9.41 | 9.54 | 82 |

**Supplementary Table S2.** Participant-level area under the curve (AUC) with 95% confidence intervals (DeLong), DeLong test *p* values, and numbers of positive and negative participants for the MSSQ and the VTC across maximum MISC thresholds (*k*) in Experiments 1 and 2. At *k* = 1 the AUC is not estimable (n.e.) because all participants reached the threshold. AUC, area under the curve; MSSQ, Motion Sickness Susceptibility Questionnaire; VTC, vestibular time constant; MISC, Misery Scale.

| *k* | Positive | Negative | VTC AUC (95% CI) | MSSQ AUC (95% CI) | DeLong *p* |
| --- | --- | --- | --- | --- | --- |
| Experiment 1 (acceleration, N = 21) | | | | | |
| 1 | 21 | 0 | n.e. | n.e. | n.e. |
| 2 | 18 | 3 | 0.65 (0.35–0.95) | 0.60 (0.32–0.89) | 0.850 |
| 3 | 17 | 4 | 0.59 (0.31–0.87) | 0.57 (0.31–0.82) | 0.908 |
| 4 | 16 | 5 | 0.60 (0.35–0.85) | 0.68 (0.42–0.93) | 0.687 |
| 5 | 13 | 8 | 0.67 (0.43–0.91) | 0.62 (0.35–0.89) | 0.751 |
| 6 | 7 | 14 | 0.75 (0.46–1.00) | 0.64 (0.39–0.89) | 0.423 |
| 7 | 6 | 15 | 0.80 (0.50–1.00) | 0.71 (0.47–0.94) | 0.437 |
| 8 | 2 | 19 | 0.92 (0.75–1.00) | 0.61 (0.35–0.86) | <0.001 |
| Experiment 2 (deceleration, N = 24) | | | | | |
| 1 | 24 | 0 | n.e. | n.e. | n.e. |
| 2 | 22 | 2 | 0.48 (0.24–0.72) | 0.72 (0.19–1.00) | 0.290 |
| 3 | 22 | 2 | 0.48 (0.24–0.72) | 0.72 (0.19–1.00) | 0.290 |
| 4 | 20 | 4 | 0.60 (0.35–0.85) | 0.68 (0.37–0.98) | 0.733 |
| 5 | 18 | 6 | 0.77 (0.55–0.98) | 0.67 (0.44–0.90) | 0.588 |
| 6 | 12 | 12 | 0.89 (0.76–1.00) | 0.60 (0.36–0.84) | 0.048 |
| 7 | 9 | 15 | 0.96 (0.88–1.00) | 0.63 (0.38–0.87) | 0.007 |
| 8 | 5 | 19 | 0.95 (0.84–1.00) | 0.81 (0.62–1.00) | 0.228 |

**Supplementary Figure S1.** Scatter plots presenting the relationships between MSSQ scores and (a) mean MISC, (b) maximum MISC, (c) cumulative MISC, and (d) last MISC scores for the full participant group (N=21) in Experiment 1 (acceleration-induced condition). MSSQ, Motion Sickness Susceptibility Questionnaire; MISC, Misery Scale.

**Supplementary Figure S2.** Scatter plots presenting the relationships between MSSQ scores and (a) mean MISC, (b) maximum MISC, (c) cumulative MISC, and (d) last MISC scores for the symptomatic subgroup (N=16) in Experiment 1 (acceleration-induced condition). MSSQ, Motion Sickness Susceptibility Questionnaire; MISC, Misery Scale.

**Supplementary Figure S3.** Scatter plots presenting the relationships between MSSQ scores and (a) mean MISC, (b) maximum MISC, (c) cumulative MISC, and (d) last MISC scores for the full participant group (N=24) in Experiment 2 (deceleration-induced condition). MSSQ, Motion Sickness Susceptibility Questionnaire; MISC, Misery Scale.

**Supplementary Figure S4.** Scatter plots presenting the relationships between MSSQ scores and (a) mean MISC, (b) maximum MISC, (c) cumulative MISC, and (d) last MISC scores for the symptomatic subgroup (N=20) in Experiment 2 (deceleration-induced condition). MSSQ, Motion Sickness Susceptibility Questionnaire; MISC, Misery Scale.

**Supplementary Figure S5.** Scatter plots presenting the relationships between VTC and (a) mean MISC, (b) maximum MISC, (c) cumulative MISC, and (d) last MISC scores for the full participant group (N=21) in Experiment 1 (acceleration-induced condition). MSSQ, Motion Sickness Susceptibility Questionnaire; MISC, Misery Scale.

**Supplementary Figure S6.** Scatter plots presenting the relationships between VTC and (a) mean MISC, (b) maximum MISC, (c) cumulative MISC, and (d) last MISC scores for the symptomatic subgroup (N=16) in Experiment 1 (acceleration-induced condition). VTC, vestibular time constant; MISC, Misery Scale.

**Supplementary Figure S7.** Scatter plots presenting the relationships between VTC and (a) mean MISC, (b) maximum MISC, (c) cumulative MISC, and (d) last MISC scores for the full participant group (N=24) in Experiment 2 (deceleration-induced condition). VTC, vestibular time constant; MISC, Misery Scale.

**Supplementary Figure S8.** Scatter plots presenting the relationships between VTC and (a) mean MISC, (b) maximum MISC, (c) cumulative MISC, and (d) last MISC scores for the symptomatic subgroup (N=20) in Experiment 2 (deceleration-induced condition). VTC, vestibular time constant; MISC, Misery Scale.

**Supplementary Figure S9.** Participant-level receiver operating characteristic (ROC) curves for the MSSQ and the VTC at each threshold of the maximum MISC score (*k* = 1–8) under the acceleration condition (Experiment 1). The *k* = 1 threshold is not estimable because all participants reached it. AUC, area under the curve; MSSQ, Motion Sickness Susceptibility Questionnaire; VTC, vestibular time constant; MISC, Misery Scale.

**Supplementary Figure S10.** Participant-level receiver operating characteristic (ROC) curves for the MSSQ and the VTC at each threshold of the maximum MISC score (*k* = 1–8) under the deceleration condition (Experiment 2). The *k* = 1 threshold is not estimable because all participants reached it. AUC, area under the curve; MSSQ, Motion Sickness Susceptibility Questionnaire; VTC, vestibular time constant; MISC, Misery Scale.
